# Recruiting Conventional Tree Architecture Models into State-of-the-Art LiDAR Mapping for Investigating Tree Growth Habits in Structure

**DOI:** 10.3389/fpls.2018.00220

**Published:** 2018-02-20

**Authors:** Yi Lin, Miao Jiang, Petri Pellikka, Janne Heiskanen

**Affiliations:** ^1^Beijing Key Lab of Spatial Information Integration and Its Applications, School of Earth and Space Sciences, Institute of Remote Sensing and GIS, Peking University, Beijing, China; ^2^Institute of Mineral Resources Research, China Metallurgical Geology Bureau, Beijing, China; ^3^Department of Geosciences and Geography, University of Helsinki, Helsinki, Finland

**Keywords:** tree growth habits, Hallé architecture model (HAM), light detection and ranging (LiDAR), tree species classification, morphological features

## Abstract

Mensuration of tree growth habits is of considerable importance for understanding forest ecosystem processes and forest biophysical responses to climate changes. However, the complexity of tree crown morphology that is typically formed after many years of growth tends to render it a non-trivial task, even for the state-of-the-art 3D forest mapping technology—light detection and ranging (LiDAR). Fortunately, botanists have deduced the large structural diversity of tree forms into only a limited number of tree architecture models, which can present *a-priori* knowledge about tree structure, growth, and other attributes for different species. This study attempted to recruit Hallé architecture models (HAMs) into LiDAR mapping to investigate tree growth habits in structure. First, following the HAM-characterized tree structure organization rules, we run the kernel procedure of tree species classification based on the LiDAR-collected point clouds using a support vector machine classifier in the leave-one-out-for-cross-validation mode. Then, the HAM corresponding to each of the classified tree species was identified based on expert knowledge, assisted by the comparison of the LiDAR-derived feature parameters. Next, the tree growth habits in structure for each of the tree species were derived from the determined HAM. In the case of four tree species growing in the boreal environment, the tests indicated that the classification accuracy reached 85.0%, and their growth habits could be derived by qualitative and quantitative means. Overall, the strategy of recruiting conventional HAMs into LiDAR mapping for investigating tree growth habits in structure was validated, thereby paving a new way for efficiently reflecting tree growth habits and projecting forest structure dynamics.

## Introduction

Trees play a fundamental role in maintaining forest ecosystems by adapting their biophysical, biochemical or physiological characteristics to the integrity of biotic and abiotic environmental influences (Saxe et al., 2001). The adaptation effects apparently show in their structures and growth habits (Creber and Chaloner, 1984). For example, the contemporary structures of trees mirror the inherent rules of their various organic components being organized; by following these rules, people estimated the function capacities of trees such as crown photosynthesis efficacy (Lovelock et al., 2006). As regards to the dynamical growth habits of trees such as their continuous or rhythmic growth modes, tree habit modeling facilitates decisions-making in advance, which is favorable for sustainable forest managements (Pretzsch et al., 2006). Hence, learning tree growth habits in structure has considerable implications for understanding from how single trees cope with diversified environmental stresses to how integral forests function in multiplex terrestrial processes (Pinard et al., 1999). This task is especially interesting for the trees growing in the boreal environment, since characterizing tree growth habits in structure in such a condition that is enduring quicker warming than global warming (Shepherd, 2016) facilitates projecting how trees will respond to the climatic scenarios in the future.

However, mensuration of tree growth habits in structure across large areas is a non-trivial task. This is due to the difficulty of measuring and modeling tree crown structures that comprise leaves and branches of varying shape forms, growth modes, and organization layouts at the fine scales. In addition, the conventional approaches, e.g., field investigation and estimation of tree growths by comparing multi-temporal canopy surface models (Yu et al., 2006), are far from enough for accurately mapping crown structure and dynamics. Therefore, new techniques with higher potentials of characterizing tree growth habits in structure are widely and urgently demanded.

Remote sensing (RS) supplies a more efficient solution plan to accomplish this task (Heinzel and Koch, 2012). The state-of-the-art RS technology—static terrestrial laser scanning (TLS), with the merits of 3D high-density sampling of objects, seems to be the best choice to date. Based on TLS data, an *L*iDAR data to tree *Architect*ure (*L-Architect*) architecture model has been designed to provide a feasible framework for quantifying the spatial distributions of tree components and explicitly describing 3D tree architectures (Côté et al., 2011). From TLS-recorded 3D points, quantitative retrievals of crown structure and foliage assemblage situations have also been tried (Yang et al., 2013). In a broader sense, TLS has also been enthusiastically attempted for deriving various structural parameters of trees, including leaf area density (Hosoi and Omasa, 2007), tree height and crown diameter (Moorthy et al., 2011; Lin et al., 2012), and tree biomass (Calders et al., 2015). However, although TLS has proved to be able to characterize tree structures well, it is difficult to derive tree growth habits directly from TLS point clouds due to the complex structures and organizations of leaves and branches. Eitel et al. (2013) placed a TLS system at a fixed location for monitoring forest plot dynamics, but the time span was too short to infer the growth habits in structure. Worse still, TLS is principally restricted by its limited coverage; consequently, TLS is more assumed to supply the reference samples for calibrating large-area forest canopy structure measurements (Côté et al., 2012).

As another technical branch of light detection and ranging (LiDAR), airborne laser scanning (ALS) that is applicable for large-scale forest mapping can solve this shortage to some extent. In retrospect, the usages of ALS for forest inventory had a longer history than TLS (Yang et al., 2013), while this history showed that applying ALS for forest inventories still needs to consider tree architectures when mapping accuracies are concerned. This is evidenced by the fact that numerous ALS-based forest researches were conducted based on presumptions of diversified tree crown archetypes, as reviewed by Calders et al. (2013). For example, Koetz et al. (2007) used a hemi-ellipsoidal tree crown archetype, and Ferraz et al. (2012) assumed an ellipsoidal one. The results indicated that geometric archetypes of tree crowns did help in forest investigations, yet the previous selections of archetypes were mostly based on the external shape outlines of tree crowns represented by 3D ALS points (Koetz et al., 2007; Ferraz et al., 2012; Calders et al., 2013). That is, almost neither geometries nor topologies of crown-internal structures such as the layouts of leaves and branches were examined, and most of the previous studies have not considered tree architectures in a real sense. This is caused by that the relatively low sampling density of airborne LiDAR is the most kernel factor restricting its performance on detailed representations of crown architectures. This adverse condition also restricts the solutions of fine-scale plant architecture modeling (Davidson et al., 2008) from playing their full roles. Although the latest ALS systems are continuously improved with their sampling densities increased, they are far from enough for characterizing crown-internal structural details (Ko et al., 2013).

Fortunately, botanists have generalized tree forms of a gigantic diversity into a limited number of tree architecture models, which facilitate sketchily giving *a-priori* knowledge of tree structure and development. Hallé et al. (1978) proposed a scheme to deduce the branch and stem architectures of tropical trees into 23 tree architecture models (hereafter called Hallé architecture models, HAMs). These HAMs have also been applied onto multiple temperate tree species (Fisher, 1984; Costes et al., 2006). As a representative set of tree architecture models, their proposal was rooted in that there are inherent modes of organizing stems and branches for different tree species, and the associated genetically-determined rules don't vary along with trees growing (Negrón et al., 2013). Tree architecture models have also been used to determine the branching forms considered in plant constructions (Barthélémy and Caraglio, 2007), e.g., based on the hidden semi-Markov models (Guédon et al., 2001) or the stochastic models (Costes and Guédon, 2002; Renton et al., 2006). HAMs have also shown their usefulness in improving the understanding of tree, canopy, and even forest properties. For example, Mutke et al. (2005) applied a Rauh HAM for characterizing the grafted stone pines (*Pinus pinea* L.) to figure out their shoot growths and bud differentiations. Feng et al. (2012) synthesized a Rauh HAM and a forest dynamics model to simulate the development of a forest stand, with the stand-level ecological/silvicultural model and the tree-level architectural representations integrated to provide the details of the individual trees at the stand level.

With the HAMs used as a linking bridge, ALS can supply more information on the structural, morphological, and biophysiological properties of trees. Theoretically, although ALS cannot make the same performance on tree structure reconstruction as TLS does, it can reveal the sketchy features such as the spatial pattern of laser points, the penetrations for different crown layers and crown integral symmetry. These three case features correspond to the external shapes of trees and crown-internal structures (i.e., leaf distributions and branching modes), which are of the potentials for deriving the appropriate HAMs. Actually, in the previous ALS-based endeavors, it has already been realized that grasping more detailed structural features of crowns can better the depth of tree understanding (Hyde et al., 2005; Wang and Glenn, 2008; Ferraz et al., 2012). With the promotion of ALS performance, particularly on the sampling density, the trend of making use of crown-internal structure is getting more and more distinct. For instance, tree genera classification has been explored with the geometric traits of branches measured with high-density ALS (Ko et al., 2013). The HAMs proposed by Hallé et al. (1978) were defined in a descriptive (qualitative) way; hence, no explicit attempts, so far, have emerged to connect these theoretical models to real ALS point clouds to derive more about tree growth habits in structure. Consequently, a question can be asked—Investigating tree growth habits in structure: using traditional HAMs, state-of-the-art ALS, or both?

To fill this gap, this study attempted to recruit the conventional tree architecture models into the state-of-the-art ALS based investigation of tree-level properties, here tree growth habits in structure. As suggested by Hallé et al. (1978), HAMs depend on tree species. Accordingly, the used strategies were to classify tree species based on the ALS-collected data, identify the HAM for each of the targeted tree species, and derive the properties of the targeted trees by referring to their related HAMs. To validate this solution plan, this work needed to implement the following three tasks: (1) converting the descriptive HAM-indicated structural traits into a set of quantitative parameters by following the characteristics in the ALS-based tree representation; (2) classifying tree species based on the proposed crown- and tree-associated feature parameters; and (3) identifying the HAM for each of the tree species to supply the information about tree growth habits in structure.

## Materials and methods

### Study area and data collection

The study area was located on the Seurasaari Island, Helsinki, Southern Finland (N60°10′52″, E24°53′4″). The island is a wooded park and has rocks, hills, wetlands, and herb-rich forests, covering approximately a total of 46 hectares. The tree species include Norway spruce (*Picea abies*), Scots pine (*Pinus sylvestris*), European rowan (*Sorbus aucuparia*), European aspen (*Populus tremula*), alder (*Alnus* sp.), birch (*Betula* sp.), English oak (*Quercus robur*), Norway maple (*Acer platanoides*), and small-leaved lime (*Tilia cordata*), in a descending order of abundance. The studied trees lie in the middle of the forest with varying understory vegetation.

The airborne LiDAR data were collected by using an Optech ALTM 3100 laser scanner (Optech Inc., Ontario, Canada) in September 2010. The flight altitude was set at ~400 m. One laser pulse triggered 1, 2, 3, or 4 returns. The density of the collected point clouds is ~10 points per m^2^, which can be classified into an intermediate point density compared with the very-high density reported by Ko et al. (2013). To acquire the reference data, the TLS data were collected with a Leica HDS6100 system (Leica Geosystem AG, Heerbrugg, Switzerland) in September 2010. HDS6100 is a 690 nm phase-based continuous-wave laser scanner with a 360° × 310° Field-of-view; its data acquisition rate is 508, 000 points per second. The distance measurement accuracy is ±2 mm at a range distance of 25 m. The point spacing is 6.3 mm at 10 m. The TLS measurements were finished in a multi-scan mode (Liang and Hyyppä, 2013), which can result in good point coverage and further supply tree structure representations as the reference data. More detailed specifications of the test settings were described by Holopainen et al. (2013). The field inspections were conducted in January 2012, and the species of the target trees were visually identified.

### Data preparation

Based on the pre-set reference markings, the ALS and TLS data were registered by using the Cyclone software (Leica Geosystem AG, Heerbrugg, Switzerland). The procedures of tree isolation and stem segmentation were interactively accomplished by using the Terrascan software (Terrasolid Oy, Helsinki, Finland), and the detailed operations of these isolation and segmentation were delineated by Holopainen et al. (2013). The stem axes of the isolated trees were determined by principal component analyses (PCA) of the extracted laser points on stems (Lehtomäki et al., 2010). The results can help calculate tree feature parameters in a uniform manner.

**Figure 1:**
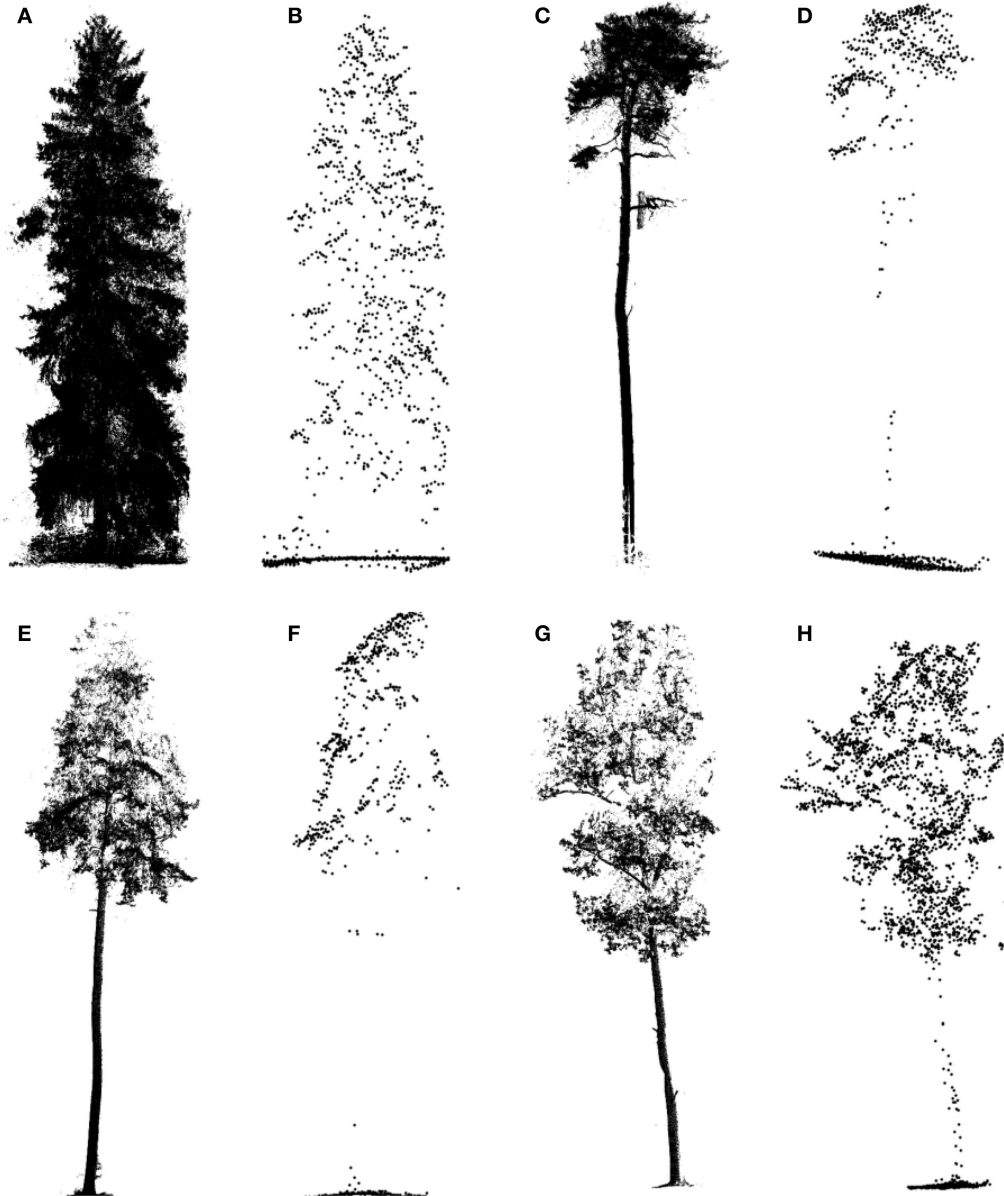
Illustrations of the sample trees represented by TLS **(A,C,E,G)** and ALS **(B,D,F,H)** data for the different tree species: *Picea abies* (PA) **(A,B)**, *Pinus sylvestris* (PS) **(C,D)**, *Populus tremula* (PT) **(E,F)**, and *Quercus robur* (QR) **(G,H)**.

After data preprocessing, a total of 40 trees with their structural representations simultaneously by the ALS and TLS data were chosen for the tests. The trees belong to four typical boreal tree species, including nine Norway spruces (*P. abies*, PA), 14 Scots pines (*P. sylvestris*, PS), 7 European aspens (*P. tremula*, PT), and 10 English oaks (*Q. robur*, QR). The specimens represented by the ALS and TLS data are illustrated in Figure 1. Their descriptive statistics in terms of tree height and crown length (i.e., crown thickness Hauglin et al., 2014) are listed in Table 1.

**Table 1:** Descriptive statistics for the sample trees.

**Tree species**	**Number**	**Tree height (m)**			**Crown length (m)**		
		**Max**	**Min**	**Mean**	**Max**	**Min**	**Mean**
*Picea abies*	9	28.38	17.60	23.68	27.38	14.02	21.82
*Pinus sylvestris*	14	23.73	16.66	21.06	18.83	5.67	11.17
*Populus tremula*	7	25.98	20.39	23.63	20.11	11.64	16.37
*Quercus robur*	10	25.94	15.17	20.21	18.94	13.81	16.69

### Schematic program

**Figure 2:**
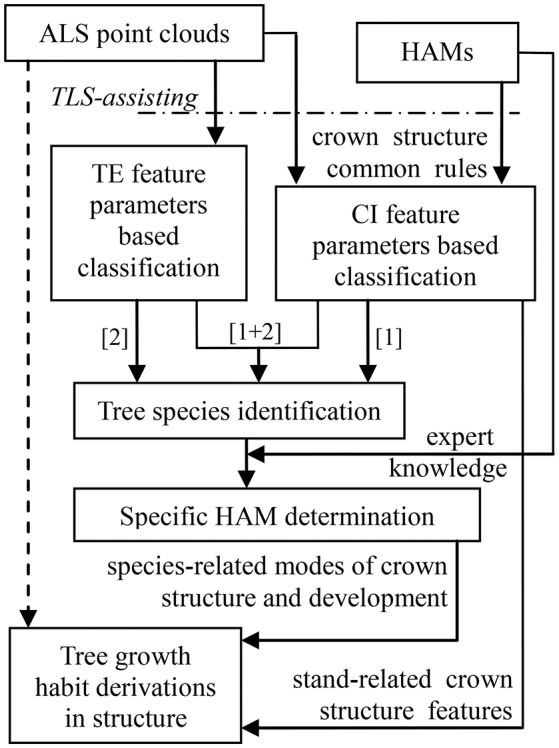
Workflow of the proposed schematic program in this study.

The schematic program of combining the HAMs and ALS was designed (Figure 2). The dashed arrow line marks the status that it is hard to directly derive tree growth habits in structure from ALS data. After all, different to TLS that is even available for tree architecture modeling at the branch scales (Côté et al., 2011), most contemporary ALS systems show low point densities. The HAMs are used to work as a bridging link to fill this technical gap. Specifically, the HAM-delineated tree structural organization rules are used to guide tree species classification; then, the HAM that can best model each tree species is identified; finally, the structural growth habits for each of the tree species of interest are derived from its associated HAM. In this schematic framework, tree species classification serves as a kernel procedure; hence, seeking how to add the accuracy of this procedure is very important.

For LiDAR-based tree species classification, a large number of approaches has been proposed, even aiming at the scenario that merely limited-size sample data are collected (Puttonen et al., 2010). Their specific methodologies have proved to work well on the corresponding ALS data (Dalponte et al., 2008; Heinzel and Koch, 2012; Ko et al., 2013) and TLS data (Lin and Herold, 2016) that are of varying conditions. However, almost all of these previous studies suggested that both distinctive feature variables and high-performance classifiers are still needed. This schematic program was aimed at these two aspects, i.e., comprehensively proposing feature parameters and classifiers. For example, the parameters capable of reflecting tree external (TE) structures such as tree height can be intuitively extracted, but the feature parameters reflecting crown internal (CI) structures such as tree symmetry need to be proposed by referring to the structural characteristics presented by the associated HAMs. The classifications based on these two kinds of parameters correspond to the routines [1] and [2] in Figure 2, respectively. Tree species classification can be deployed on the two sets of feature parameters separately or together ([1]+[2]) to enhance the overall accuracy. The following sub-Section Tree Species Classification specifically describes how to derive feature parameters from the HAMs and the selected classifier appropriate for the associated classification scenario in the present study. Next, the sub-section HAM Identification and Information Derivation delineates how to determine the related HAMs, via referring to the expert knowledge supplied by the potential HAMs, and derive tree growth habits in structure.

### Tree species classification

#### TLS-assisted analysis of HAM signatures

**Figure 3:**
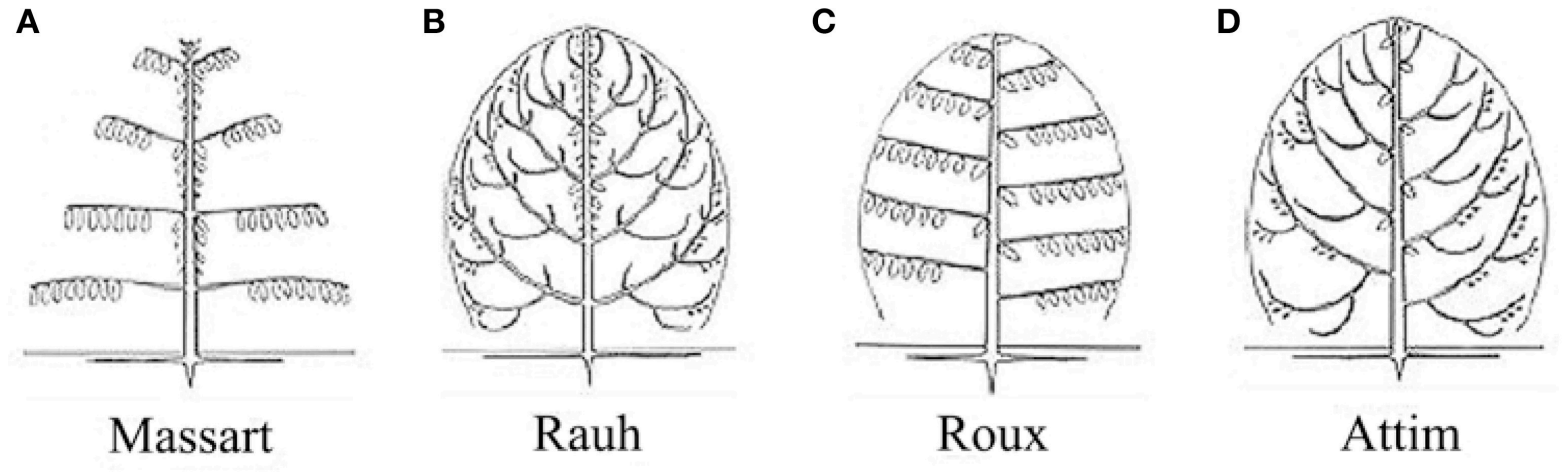
Illustrations of the HAMs proposed by Hallé et al. (1978): **(A)** Massart, **(B)** Rauh, **(C)** Roux, and **(D)** Attim.

For the four tree species, their HAMs were visually interpreted in advance, via referring to Prusinkiewicz and Remphrey (2000), in order to obtain the ground-truth data that were used to test the performance of the proposed schematic program. The manually-identified HAMs are demonstrated in Figure 3. The Massart model shows plagiotropic structure, with the main branches being in whorls and rhythmic growths. The Rauh model shows different orthotropic structure, with its main branches being morphogenetically equivalent to its trunk, all in a rhythmic growth mode. The Roux model demonstrates plagiotropic, monopodial, and non-phyllomorphic structure and continuous growths. The Attim model exhibits orthotropic structure and more or less continuous growths, also with its main branches being morphogenetically equivalent to its trunk. More specific information about tree growth habits in structure can refer to the relevant literature (Hallé et al., 1978; Fisher, 1984; Costes et al., 2006). Given that the focus of this study was on examining the proposed schematic program rather than on revealing more growth habits of the sample trees, the above-listed structures such as plagiotropic structure and continuous growth were referred to for proposing more appropriate feature parameters for tree species classification and deriving tree growth habits in structure.

#### Proposal and derivation of tree structure feature parameters

The performance on exploiting tree structure growth habits is largely dependent on whether the assumed feature parameters are suitable for tree species classification. However, none of the previous studies have generalized each of the 23 HAMs into a set of distinguished feature parameters. To fill this gap, the first work was to propose feature parameters effective for operating the step of tree species classification, with the HAM-indicated descriptive signatures and the characteristics of ALS-based tree representation considered in a comprehensive way. That is, the derived tree structure feature parameters from the ALS-based tree representations need to characterize the marked differences of orthotropic vs. plagiotropic branching and rhythmic vs. continuous growth reflected by the four models as much as possible.

First, the CI structure feature parameters were proposed. For different branching patterns within crowns, the related ALS-collected point clouds demonstrate different spatial-distribution modes. For example, the orthotropic branches often correspond to a symmetric distribution of point clusters that represent branches, but the plagiotropic branches more often mean high laser penetration in trees. Such knowledge comprises the rules linking between the structural features and the HAMs. In accordance to this rule set, the HAM-associated CI structure parameters were extracted by applying the 8-segments framework developed by Lin and Hyyppä (2016). Next, a total of 13 CI parameters were extracted, with their derivations from the specific characteristics of the HAMs and their quantitative definitions listed in Table 2. For instance, the fourth parameter (P4) is defined as the largest voxel density within 1 m super-voxels to reflect the HAM situation of leaf clustering. These parameters can allude to different branching and leaf distribution modes, which are characteristic for different HAMs.

**Table 2:** CI feature parameters derived from the ALS point clouds by referring to the HAMs.

**Abbr**	**Definition**	**HAM-rule**	**Formula**
P1	Ratio between the height of the equivalent centers for the voxels within each profile and crown length (average for 8 profiles)	Horizontal branch arrangement	$\text{P}1=\frac{\sum_{8}\left(\sum_{i=1}^{n}H_{G_{i}}/n\right)}{8\cdot L_{c}}$
P2	Ratio between the radius of the equivalent centers for the voxels within 8 profiles and crown radius (average for 8 profiles)	Vertical branch arrangement	$\text{P}2=\frac{\sum_{8}\left(\sum_{i=1}^{n}R_{{\text{G}}_{i}}/n\right)}{8\cdot R_{\text{c}}}$
P3	Ratio between the area of the below 1/3 laser points and crown base area	Old branches longer or not	$\text{P}3=A_{\text{down}\frac{1}{3}\text{pts}}/A_{\text{Base}}$
P4	Largest voxel density within 1 m super-voxels	Leaf clustering	$\text{P}4=\max\left(N_{G_{\text{1m}}}\right)$
P5	Standard deviation of P1 for 8 profiles	Consistency	$\text{P}5=\text{std}\left(\text{P}1_{\text{i}}{|}_{\text{i}}=1,...,8\right)$
P6	Standard deviation of P2 for 8 profiles	Consistency	$\text{P}6=\text{std}\left(\text{P}2_{\text{i}}{|}_{\text{i}}=1,...,8\right)$
P7	Ratio between the voxels of stem space and all voxels (space: 1/3 height, 1/2 crown diameter)	Branching density	$\text{P}7=\frac{\sum N_{{\text{G}}_{\text{down}\frac{1}{3}\text{crown​​}\&\text{​​within}\frac{1}{2}\text{crown}}}}{\sum N_{{\text{G}}_{\text{crown}}}}$
P8	Ratio between the height of the equivalent centers for the whole crown and crown length	Main part of growth	$\text{P}8=\frac{\sum_{8}\left(\sum_{i=1}^{n}H_{{\text{G}}_{\text{i}}}/n\right)}{8\cdot L_{c}}$
P9	Standard deviation of voxels for crown layers	Consistency	$\text{P}9=\text{std}\left(N_{{\text{G}}_{i}|\text{Layer}=1,\cdots ,V}\right)$
P10	Ratio of the sum of the difference between adjacent crown layers and all voxels	Vertical consistency	$\text{P}10=\frac{\sum_{i=1}^{8}\text{Diff}\left({\text{S}}_{\text{i}},{\text{S}}_{\text{i}+1}\right)}{\sum \text{Diff}\left({\text{G}}_{\text{i}},{\text{G}}_{\text{j}}\right)}$
P11	Ratio between the alpha volume (0.5 m) and the volume of the convex hull of crown	Branching dependence	$\text{P}11=\frac{\text{convexhull}({\text{P}}_{\text{i}}{\;}_{|\text{t}=0.5\text{m}})}{\text{convexhull}({\text{P}}_{\text{i}}{\;}_{|\text{t}=\infty })}$
P12	Ratio between the similarity of two opposite and two adjacent profiles	Branching symmetry	$\text{P}12=\frac{\sum_{\text{i}=1}^{4}\text{Cor}\left({\text{S}}_{\text{i}},{\text{S}}_{\text{i}+4}\right)}{\sum_{\text{i}=1}^{8}\text{Cor}\left({\text{S}}_{\text{i}},{\text{S}}_{\text{i}+1}\right)}$
P13	P5/P6	Consistency	P13=P5/P6

*Where L_c_ is crown length, S_i_ is the ith segment, N_G_i__ is the point number within the ith voxel, H_G_i__ is the height of the ith voxel, all of the voxels at the same height compose the related crown layer, R_G_i__ is the radius of the ith voxel, and n indicates the total number of the voxels for each tree*.

Then, the TE structure feature parameters were also proposed. The procedures of deriving the HAM-related TE structural features followed the workflow proposed by Lin and Herold (2016). In addition to the common structural parameters (Lin and Herold, 2016), additional structural parameters were defined (Table 3), by seeking the corresponding more robust parameters or by combining two common parameters. For the first case, the diameter of the circle with area equal to the crown cover (D_EA_) was defined, which can reduce the effects of crown shape variability. For the second case, the ratios between a couple of feature parameters and tree height were also examined, as shown by the parameter of LcHt (Table 3). This operation can handle the influences of varying tree ages on tree species classification. The specific 10 TE parameters can also characterize HAMs, and their combinations are of the potential for reflecting the structural differences between the four tree species.

**Table 3:** TE feature parameters derived from the ALS point clouds by referring to the HAMs.

**Abbr**.	**Definition**	**HAM-rule**	**Formula**
Ht	Tree height (Ht)	Main part of growth	${\text{H}}_{\text{t}}=\max(H_{\text{i}})-\text{DTM}$
LcHt	Ratio between crown length (Lc) and tree height (Ht)	Consistency	$\text{LcHt}=L_{\text{c}}/H_{\text{t}}$
D_EA_	Area-equivalent crown diameter (D_EA_)	Horizontal branch arrangement	${\text{D}}_{\text{EA}}=2\cdot \text{sqrt}\left(A_{\text{Base}}\right)$
Alpha	Alpha of Gaussian fitting in crown ellipsoid modeling	Branching symmetry	$\alpha |\exp\left(-\frac{{\left(x-u\right)}^{2}}{2\alpha^{2}}\right)$
LcD_EA_	Ratio between crown length (Lc) and crown diameter	Branching symmetry	${\text{LcD}}_{\text{EA}}=L_{\text{c}}/D_{\text{EA}}$
LllsLhls	Ratio between (tree height-LLS) and (tree height-HLS)	Main part of growth	$\text{LllsLhls}=\left(H_{\text{t}}-H_{\text{LLS}}\right)/\left(H_{\text{t}}-H_{\text{HLS}}\right)$
LsLcs	Ratio between LS and LCS	Main part of growth	$\text{LsLcs}=L_{\text{s}}/L_{\text{cs}}$
Gc	Mean height for all of the voxels	Vertical branch arrangement	$\text{Gc}=\sum_{i=1}^{n}H_{{\text{G}}_{i}}/n$
P_L_	Laser penetration (P_L_) into crown	Vertical branch arrangement	$P_{\text{L}}=\frac{1}{2}\cdot \left(\frac{\sum \left(n_{{\text{G}}_{\text{XZ}}}\times A_{\text{G}}\right)}{A_{\text{CV}}^{\text{XZ}}}+\frac{\sum \left(n_{{\text{G}}_{\text{YZ}}}\times A_{\text{G}}\right)}{A_{\text{CV}}^{\text{YZ}}}\right)$
LAI	Leaf area index (LAI) for all of the voxels	Leaf clustering	$LAI=\sum \left(n_{{\text{G}}_{\text{XY}}}\times A_{\text{G}}\right)/A_{\text{CV}}^{\text{XY}}$

*Where L_c_ is crown length, A_Base_ indicates the crown base area, H_HLS_ is the height of the highest branch within crown lower surface (HLS), H_LLS_ is the height of the lowest branch within crown lower surface (LLS), L_s_ is the longest spread of crown cover (LS), L_cs_ is the longest cross-spread of crown cover (LCS), H_G_i__ is the height of the ith voxel, A_G_ is the side area of each voxel, n_G_XZ__, n_G_YZ__ and n_G_XY__ each means the number of voxels in the XZ, YZ, and XY projection plane, $A_{CV}^{XZ}$, $A_{CV}^{YZ}$ and $A_{CV}^{XY}$ each means the area of the convex hull of the voxels in the XZ, YZ, and XY projection plane, respectively, and n indicates the total number of the voxels for each tree. For each tree, the variable of DTM (digital terrain model) relates to the height of the location relating to its root*.

Then, the performance about the derivation of the CI and TE feature parameters was assessed. Tree height (TH) and diameter at breast height (DBH) were deemed as the representative variables. After correlation analysis of the estimated and reference TH sets, the performance of the derivations was quantified in terms of the coefficient of determination (R^2^) and root mean squared error (RMSE), which are defined as

(1) $$\begin{array}{r} R^{2}=1-\left(\sum {\left(d_{i}-{\hat{d}}_{i}\right)}^{2}\right)/\left(\sum {\left(d_{i}-\bar{d}\right)}^{2}\right), \end{array}$$

(2) $$RMSE=\sqrt{\sum^{\text{​}}{\left(d_{i}-d_{i}^{R}\right)}^{2}/n},$$

where *d*_*i*_ is the estimated TH value for the *i*th tree; $d_{i}^{\text{R}}$ is its reference TH value; their difference $\left(d_{i}-d_{i}^{\text{R}}\right)$ is termed as TH deviation; ${\hat{d}}_{i}$ is the TH value estimated by following the estimation-reference TH regression relationship revealed by correlation analysis; and $\bar{d}=\sum d_{i}/n$.

#### Classification implementation

Before the classification, a non-parametric test was manipulated to evaluate the distinctions between the four tree species, in terms of all of the calculated CI and TE feature parameters. The significance of the differences between the four tree species was handled through conducting the multivariate analysis of variance (MANOVA) (http://www.statsoft.com/Textbook/ANOVA-MANOVA#multivariate). The specific operation was run in the Data Processing System software environment (Tang and Zhang, 2013), with the resulting *p*-values used to characterize the differences.

The classification of the four tree species was based on LIBSVM, i.e., a support vector machine (SVM) package (Melgani and Bruzzone, 2004; Pfeifer and Briese, 2007; Dalponte et al., 2008; Chang and Lin, 2014). The sample trees were classified by using the SVM classifier in a leave-one-out cross-validation (LOOCV) way (Lin and Herold, 2016; Lin and Hyyppä, 2016). Specifically, each tree was classified with the SVM classifier that was trained based on the remaining trees. The assessment of their accuracies was operated in terms of recall and precision, i.e., for any tree species, the ratio between the number of the correctly-classified trees and the number of its trees and the ratio between the number of the correctly-classified trees and the number of the classified-into-that-species trees, respectively.

To seek the optimal classification result, the LOOCV-SVM-based classification was deployed on all of the cases of combining the CI and TE feature parameters. The numbers of the cases that combine 1 to 13 CI feature parameters are 13, 78, 286, 715, 1287, 1716, 1716, 1287, 715, 286, 78, 13, and 1, respectively, and the numbers of the cases that combine 1 to 11 TE feature parameters are 11, 55, 165, 330, 462, 462, 330, 165, 55, 11, and 1, respectively. The resulting recalls and precisions were compared and the optimal accuracies were achieved. The CI and TE parameters corresponding to their optimal results (termed as CI_Opt_ and TE_Opt_ variables) were combined together as the input variables (equivalent to the case of [1]+[2] in the schematic program) to explore the best classification result. In addition to the recall and precision, Cohen's kappa coefficient (κ) (Cohen, 1960) was calculated to assess the performance of the classifications individually based on the CI, TE or the combination of the CI_Opt_ and TE_Opt_ feature parameters. κ measures the inter-rater agreements between categorical items. As a more robust measure than the simple percent agreement calculations like recall and precision, κ also considers the agreement occurring by chance.

### HAM identification and information derivation

#### HAM determination and qualitative information retrieval

After tree species classification, two best-performing parameters were extracted. Their related feature parameters for HAM identification included CI alone, TE alone, or the combination of CI and TE. With the assistance of expert knowledge about the HAMs in terms of these feature parameters, the distribution of CI and TE value-pairs (i.e., the combination of CI and TE) was assumed to accurately determine the HAM corresponding to each of the four tree species. Specifically, the scatterplots of those calculated value-pairs were divided into four local quadrants, in accordance to their relative ranges for the four HAMs, e.g., the Massart model relating to low P1 values and low P13 values, the Rauh model to high P1 and low P13, the Roux model to low P1 and high P13, and the Attim model to high P1 and high P13. Next, the HAM for each group of the classified trees was identified according to its related center of the value-pairs lying in which one of the local quadrants. By this means, the influences caused by some special specimens can be reduced. Based on the determined HAMs, the knowledge about tree growth habits in structure can be derived, and the specific operations are listed as follows.

#### Quantitative information derivation and assessment

The feature parameters proposed for HAMs determination can also be assumed as the quantitative indicators of tree growth habits in structure. After the steps of tree species classification and HAMs identification, the derived feature parameters based on the classified specimens, in an isolated or integrated manner, were used to supply the information on the quantitative characteristics of tree growth habits.

The mis-classified or -identified specimens, no doubt, may introduce errors into the derived feature parameters; hence, the accuracies of the derived parameters need to be quantitatively assessed. Given that the objective of this study was to investigate tree growth habits in structure at forest plot or stand rather than individual tree levels, statistics based on Gaussian distribution were used to generate a limited number of indices, in favor of efficient accuracy assessment. For each of the prescribed feature parameter, its derived values for all of the specimens of each classified species were fitted to a Gaussian distribution function. The resulting expectation (μ) and standard deviation (σ) were compared to the derivations from the ground-reference data, and the numerical divergence was used to quantitatively evaluate the performance of the proposed method for retrievals of tree growth habits in structure.

## Results

### ALS-based tree characterization

**Figure 4:**
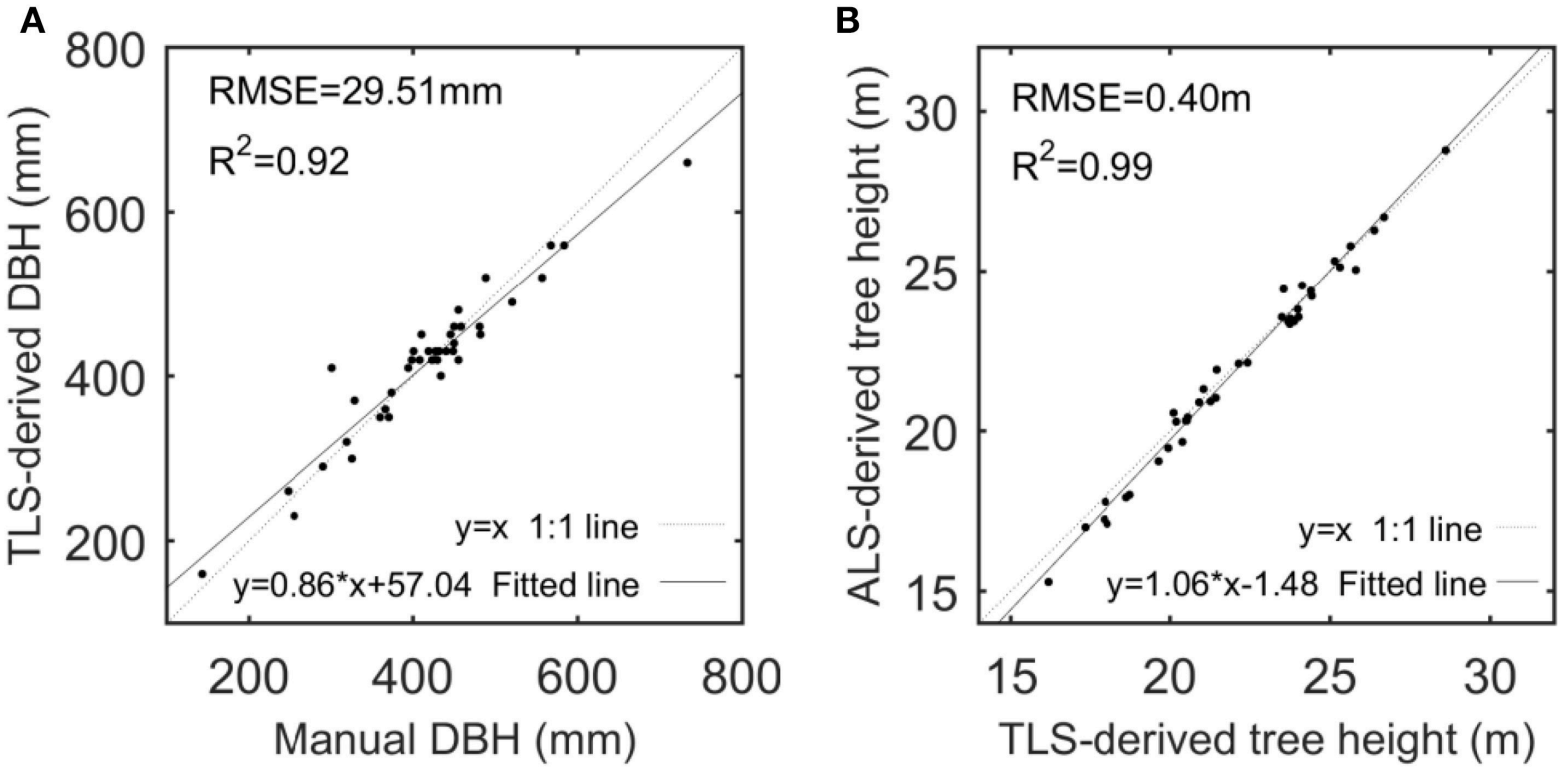
Scatterplots of TLS-derived and manually-measured DBHs **(A)** and of ALS- and TLS-derived tree heights **(B)**, with their linear models fitted by linear regression analysis.

Given that TH and DBH were selected as the representative variables to assess the derivations of the CI and TE feature parameters, their derivations from the ALS and TLS data were examined. The performance of TLS-based DBH derivations and ALS-based TH estimations are shown in Figure 4, with high-value R^2^ and low-value RMSEs but with linearly-varying estimation biases at different value ranges that are principally caused by the systematic errors of the scanning systems. The comparison between the TLS-derived and manually-measured DBHs can testify the capability of the used TLS for detailed tree representation, and the comparison between the ALS- and TLS-derived THs can verify the availability of the ALS for tree characterization. After all, TLS has been used for collecting the ground-truth data (Côté et al., 2012), and ALS data can derive other feature parameters like DBH via allometric relations (Hauglin et al., 2014; Calders et al., 2015). More assessments of the derivations of other structural feature parameters can refer to Holopainen et al. (2013). All of the results validated the premier of this study, i.e., the used ALS and TLS are applicable for characterizing the basic structures of the sample trees.

### Tree species classification

We first derived the proposed CI feature parameters (Figure S1), based on which the MANOVA *p*-value was calculated to be 0.0001 (Table S1). This meant significant differences exist between the four tree species, but the CI-based classification results (Figure S1) told that using any one of the 13 CI parameters individually cannot fulfill distinguishing the four tree species. Then, classifications were conducted for all of the cases of combining 1–13 CI parameters (Figure S2), which relates to the operations of the routine [1] marked in Figure 2. The overall performance firstly went better and then leveled off. The highest classification accuracy is 82.5% (κ = 0.794), in the case of inputting the five CI parameters—P3, P4, P6, P7, and P8—as the dependent variables into the classification (Table S2).

Then, the TE parameters were derived (Figure S3), and their related MANOVA *p*-value was 0.0001 (Table S3). The classifications for all of the cases of combining 1–10 TE structure parameters were carried out (Figure S4), which corresponds to the routine [2] in Figure 2. The highest classification accuracy is 85.0% (Table S4), in the case of combining the LcHt, LcD_EA_, Gc, D_EA_, and σ TE parameters (κ = 0.764).

**Figure 5:**
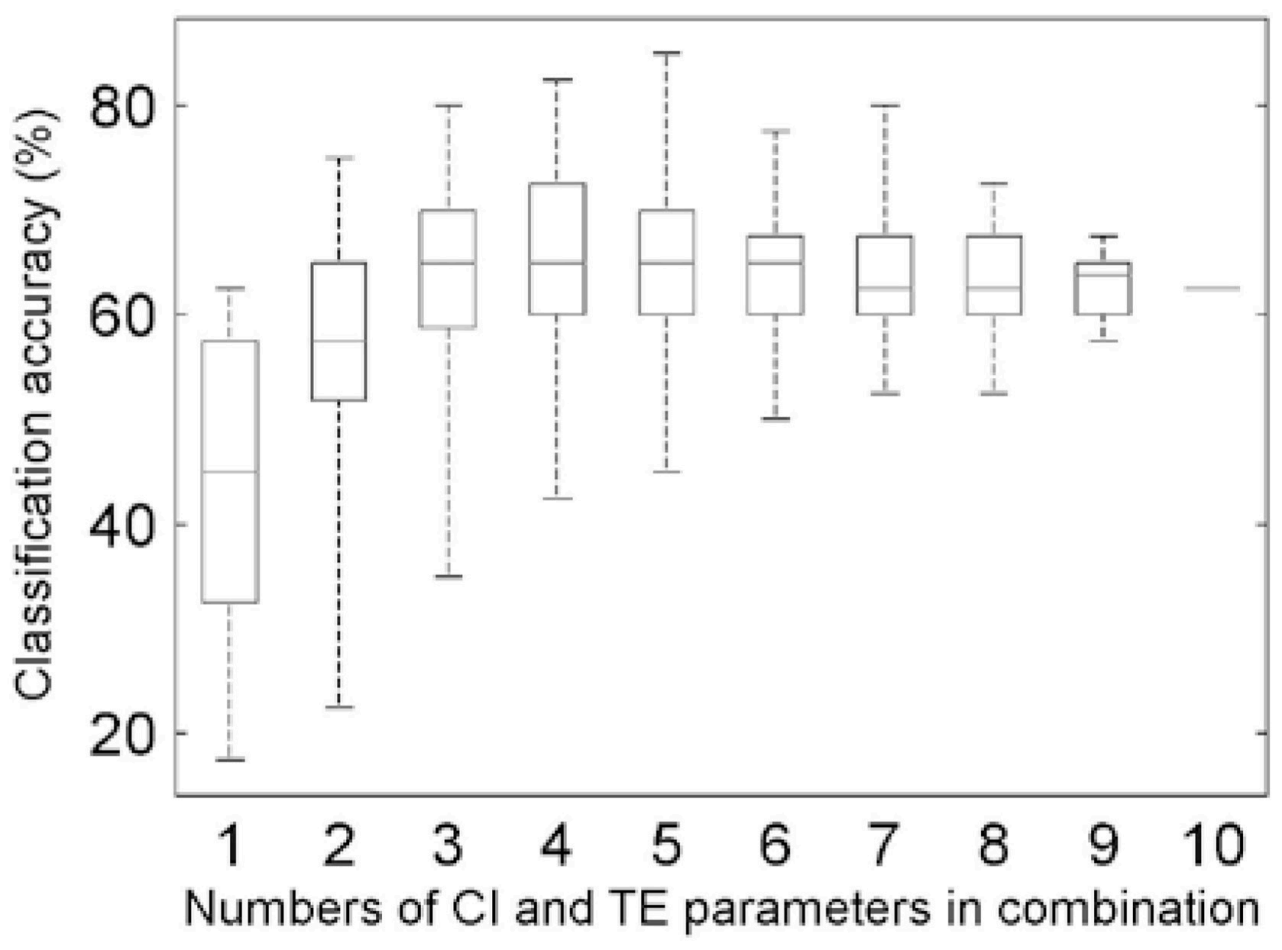
Boxplots of the overall accuracies of the classifications for different combinations of the five CI_Opt_ and five TE_Opt_ feature parameters.

The combinations of the above-sought CI_Opt_ and TE_Opt_ parameters were input to implement the classification (MANOVA *p*-value = 0.0001; Table S5). The accuracies for all of the cases of combining the optimal five CI parameters and the optimal five TE parameters were calculated (Figure 5), and this step relates to the routine [1]+[2] in Figure 2. The highest accuracy is 85.0%, which corresponds to the optimal case of TE-based classification (κ = 0.794; Table S6). Further, for the task of searching two feature parameters to determine the appropriate HAMs for the four tree species, the best case of combining the CI-typed parameters of P4 and P7 was found, exhibiting the highest classification accuracy of 75.0% (κ = 0.658).

### HAM determination and information retrieval

#### Qualitative derivation

**Figure 6:**
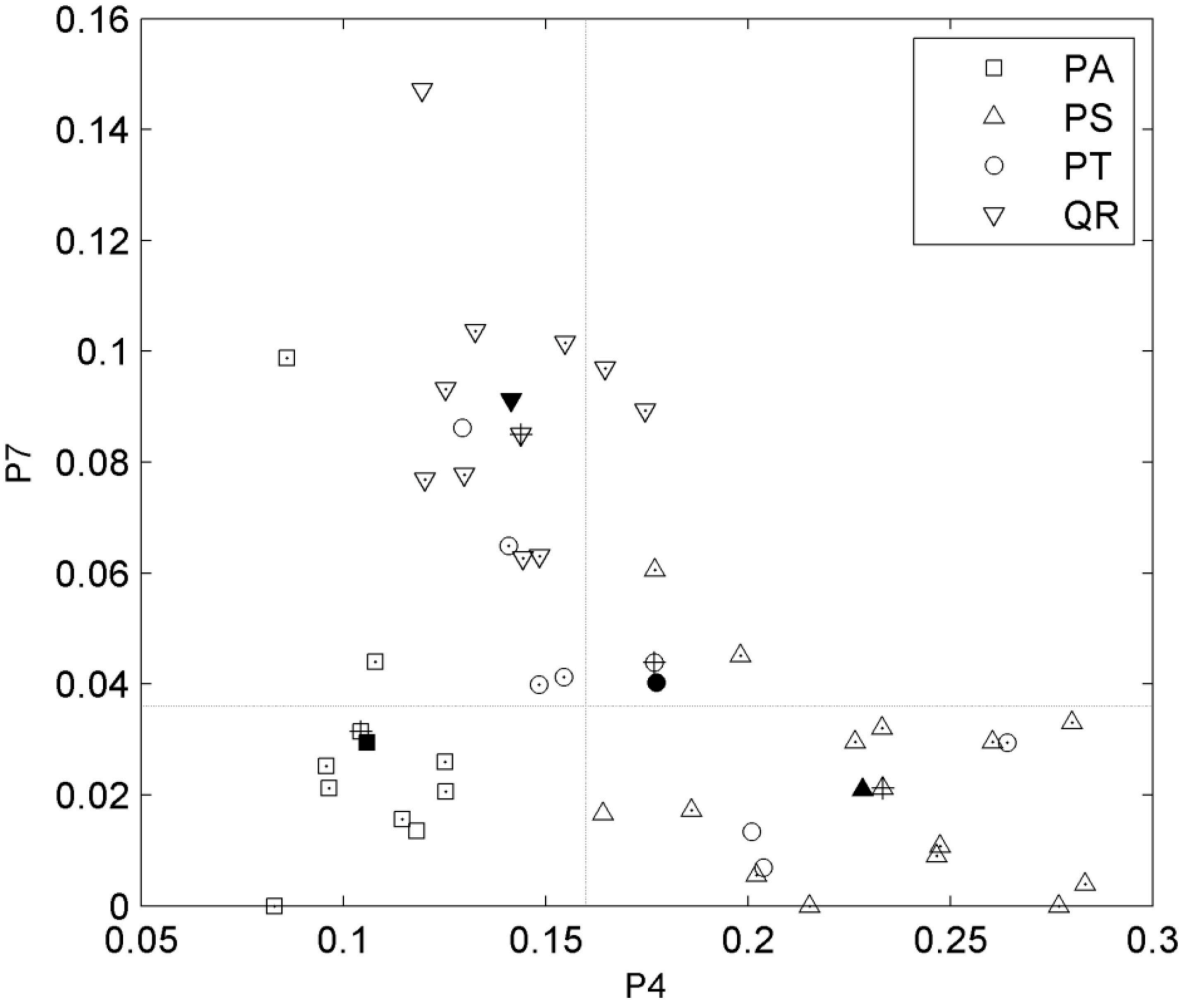
Scatterplots of the P4 and P7 feature parameters for the four tree species and their value-pair distributions for determining the related HAMs. Markers with points indicate the correctly-classified tree specimens; markers filled in black indicate the centers of the parameter-pair distributions for all tree specimens; markers with pluses indicate the centers of the parameter-pair distributions for the correctly-classified tree specimens; and two dash lines segment the four local quadrants.

After tree species classifications, the HAM for each of the four tree species was identified based on the expert knowledge, assisted by referring to the distributions of the two P4 and P7 variables in pairs (Figure 6). Specifically, for the four tree species, the centers of their P4–P7 pair distributions were derived, and a comparison of these centers to the opposites calculated by referring to the manually-trained crown-HAM links was operated to determine the HAM for each of the four tree species. The links include that the Massart model relates to low P4 and low P7 values (corresponding to the PA species), the Rauh model exhibits high P4 and low P7 (PS), the Roux model shows moderate P4 and low P7 (PT), and the Attim model presents low P4 and high P7 (QR). By this means, the *a-priori* knowledge about tree growth habits in structure was acquired for the four tree species (Table 4).

**Table 4:** Tree growth habits in structure derived for the four tree species.

**Tree specimens**	**Trunk**	**Branches**	**Growth**
*Pinus sylvestris*	Monopodial, indeterminate	Plagiotropic, (main branches) produced in whorls	Rhythmic
*Populus tremula*	Monopodial	Orthotropic, morphogenetically equivalent to the trunk	Rhythmic
*Quercus robur*	Monopodial, indeterminate	Plagiotropic, monopodial, non-phyllomorphic	Continuous
*Picea abies*	Monopodial	Orthotropic, morphogenetically equivalent to the trunk	More or less continuous

#### Quantitative derivation

**Figure 7:**
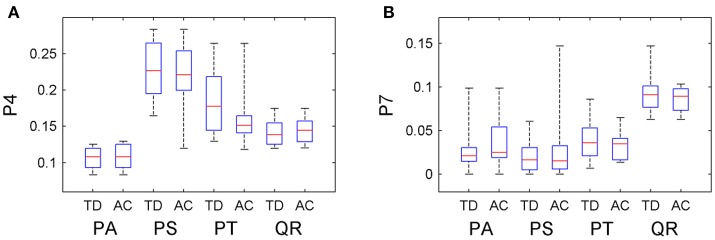
Boxplots of the **(A)** P4 and **(B)** P7 CI parameter values after the classification and HAM identification (AC), compared with their values derived from the ground-truth data (TD).

The information about tree growth habits in structure, further, was quantitatively derived, as illustrated by the boxplots of the derived parameters P4 and P7 (Figure 7). The biases between the boxplots show the distinctions between the four tree species. For each tree species, the derived parameters are approximate to their values from the ground-truth data, as evidenced by their comparative statistics (Table 5). Specifically, their absolute deviations (|Δμ|) of the expectations of the fitted Gaussian distribution before and after the proposed tree species classification and HAM identification are <7% of the actual values for any of the four tree species, and their absolute deviations (|Δσ|) of the standard deviations (σ) are all <16% of the reference values. These results suggested that based on the proposed method, the quantitative characteristics about tree growth habits in structure for the four tree species can be derived for the trees at plot or stand scales.

**Table 5:** Derivations of the (a) P4 and (b) P7 CI parameter (DP) values after the classification and HAM identification, compared with their values in the ground-truth (GT) data.

		**DP**		**GT**		**Ratio (%)**	
		**μ**	**σ**	**μ**	**σ**	**Δμ**	**Δσ**
P4	PA	0.1058	0.0161	0.1071	0.0176	1.30	1.50
	PS	0.2280	0.0409	0.2224	0.0437	−2.31	1.12
	PT	0.1844	0.0485	0.1650	0.0510	−6.65	0.83
	QR	0.1415	0.0191	0.1439	0.0186	2.12	−0.49
P7	PA	0.0294	0.0285	0.0375	0.0333	4.72	2.82
	PS	0.0201	0.0185	0.0277	0.0362	6.89	15.89
	PT	0.0393	0.0260	0.0343	0.0189	−3.19	−4.55
	QR	0.0912	0.0245	0.0850	0.0156	−4.22	−6.06

*More information is listed in the Supplementary Data file*.

## Discussion and conclusion

The results of the case study based on the four tree species growing in the boreal environment validated the hypothesis that the recruitment of conventional descriptive tree architecture models such as the HAMs into the state-of-the-art ALS-based forest mapping facilitates more efficiently deriving the typically hard-to-discern tree growth habits in structure. The contributions of this study are 2-fold. With ALS data used as a bridge, the application range of the HAMs can be expanded to the boreal forests; meanwhile, with the HAMs used as a link, the application discipline of ALS can be extended to tree growth habits in structure. In other words, the answer to the question posed in the Introduction, no doubt, is “both.” Overall, this study developed a novel pan-solution strategy, i.e., by recruiting the previously-established tree architectural, physiological, and/or biophysichemical models into the cutting-edge forest mapping technologies, for more efficiently understanding various forest growth habits, which facilitate investigating forest ecologies and projecting forest dynamics and processes under climate changes.

### Application extending

First, the implications of this study require more elucidation, because the results in Table 5 may be misleading, i.e., the results appear simple. Theoretically, the HAM related to each individual tree can be identified by the associated experts in the field (Negrón et al., 2013), and in such a sense, the development of a complicated method as shown in this study seems unnecessary. This reasoning, however, is only rooted in the results that were derived from the limited number of sample trees, without taking the broader-sense context of this study into account. This study tried to advance the field of forest mapping based on ALS, which generally can cover large areas (Ferraz et al., 2012). In this situation, manual determination of the HAM for each single tree is unadvisable. Besides, the concerned targets in practical ALS-based forest mapping tend to be tree communities, seldom single trees (Koetz et al., 2007). The HAMs can give descriptive “plagiotropic or not,” instead of quantitative “plagiotropic degree,” and they are useful for learning the whole structural traits of communities. Hence, the combination of ALS and the HAMs can help people to have a sketchy understanding of tree structural attributes for forest stands; namely, the first implication of this work is to present a solution of reflecting large-scale properties of forest dynamics, further advancing studies on plant geography and spatial ecology.

Then, for each specimen of the four tree species measured by the ALS, its HAM identification means not only grasping its growth habits in structure but also learning the rules for its symbolic delineation and graphical representation (Prusinkiewicz and Remphrey, 2000). Specifically, the proposed feature parameters can be adopted as the geometric/topologic restrictions to briefly determine the sequencing of apices and the configuration of branches; then, tree geometric models can be derived from ALS data, a difficult task for long but of extensive interest (Calders et al., 2013). The developed tree geometric models can be effectively in favor of the simulation-based analyses of forest-environment interaction effects (Lovelock et al., 2006). The botanic knowledge derived from the HAMs, further, can help to push forward the stage of tree geometric modeling to tree comprehensive biophysical modeling for better understanding forest physiologies.

### Influence analysis

The performance of the proposed method is restricted to many influence factors, and analyses indicated that there are three kinds of primary ones. First, the calculated values of the proposed feature parameters were unavoidably attached with errors. The sources of these errors may involve biased geometric modeling, incomplete structure representations, and uncertainties of parameter derivations. When the errors are large enough, such feature parameters may play negative roles in tree species classification (Lin and Herold, 2016). This limitation is evidenced by the maximum total accuracy, which initially improved and then leveled off, as indicated by the top whiskers of the boxes in Figure 5. In other words, this effect occurs in the cases involving the CI and TE parameters separately (Supplementary Figures S2 and S4) and their optimal-accuracy-related combination (Figure 5), respectively.

Second, the proposed feature parameters maybe cannot efficiently reflect some particular features of the tree species of interest. This influence factor is exemplified by the *P. tremula* trees, which showed relatively lower accuracies in all of the three steps of determining their HAMs in comparison to the other three tree species. In fact, as illustrated in Figure 1, the ALS-represented morphologies of the *P. tremula* tree and the *Q. robur* tree are similar, and it is difficult to distinguish them even by visual interpretation. This just verifies the hypothesis that the feature parameters proposed in this study may better present the characteristics of *Q. robur* than *P. tremula*.

Third, one tree species may not be strictly related to one HAM. At its different growth stages, a tree may satisfy different HAMs. Some tree species show different developments in HAMs during their ontogeny (Millet et al., 1998). Some trees may behave as satisfying multiple HAMs. Although this diversity issue can be somehow handled in the context of ALS-based large-area forest mapping as mentioned above, errors may be introduced into the results of tree species classification. This suggests that this problem may limit extending the application range of the proposed method; in addition, in the following studies more attention shall be paid onto the CI feature parameters, which were proposed by following the characteristics of HAMs.

### Potential improvements

Note that although the derivations were on the basis that the used ALS system can represent trees in a relatively complete manner (as illustrated in Figure 1), the proposed CI and TE feature parameters are far from enough for representing the real structures of the targeted trees, let alone the scenarios of other tree species and other HAMs regarded. Compared to the parameters considered in this study, multiple other structural feature parameters can be extracted by exploring other morphological or topological features of the trees. For instance, the spatial distribution mode of the primary branch centers (Lin and Herold, 2016) can be extracted to supply more information. Simultaneously, the proposal of other structural parameters needs also to regard the characteristics of ALS-based tree representations. When a crown is dense, less information is available from the related trunk (Puttonen et al., 2010); therefore, the parameters derived from such trunks cannot be directly compared in some cases. With such detailed influence factors as many as possible considered, the results will be improved. In addition, strengthening the automatic degree in deriving these feature parameters is highlighted.

The proposed approach for identification of the HAMs for different tree species did help to exploit the unreadily-observable tree growth habits in structure hidden in the ALS-collected point clouds, but this approach is still principally under qualitative interpretation. In other words, the derived knowledge of tree growth habits cannot be quantitatively used for precision managements of forests. Subsequent studies need to emphasize how to propose new feature parameters and to attempt other kinds of tree architecture models (e.g., Weber and Penn, 1995) for quantitative characterization of tree growth habits.

The used ALS system is a currently-pervasive airborne LiDAR, and its sampling density can reflect the main-stream laser scanning performance. Hence, the proposed method shall be highly applicable for practical applications. Nevertheless, many ALS systems produced at earlier times are still in use, and consequently, the sensitivity of the proposed method in response to laser point density needs to be further examined in the subsequent work, e.g., via checking its performance based on the ALS data of different sampling densities. The feasibility of the proposed method for different tree ages also needs to be examined, e.g., via applying it to the stands at varying growth stages and comparing its performance relative to different morphologies of trees.

## Author contributions

YL: designed this study and developed the method; MJ: made the data analysis; PP: analyzed the results and revised the manuscript; JH: reviewed and revised the manuscript.

### Conflict of interest statement

The authors declare that the research was conducted in the absence of any commercial or financial relationships that could be construed as a potential conflict of interest.
