# Supplementary material for: Recruiting Conventional Tree Architecture Models into State-of-the-Art LiDAR Mapping for Investigating Tree Growth Habits in Structure
Source: Front Plant Sci. 2018 Feb 20;9:220. doi: 10.3389/fpls.2018.00220 (PMC5826307; doi:10.3389/fpls.2018.00220)
Supplement: Supplementary file 1 [file Table1.DOCX]

**Recruiting conventional tree architecture models into state-of-the-art LiDAR mapping for investigating tree growth habits in structure**

Yi Lin ^1,^ *, Miao Jiang ^2^, Petri Pellikka ^3^, Janne Heiskanen ^3^

^1^ Institute of Remote Sensing and GIS, Beijing Key Lab of Spatial Information Integration and Its Applications, School of Earth and Space Sciences, Peking University, 100871 Beijing, China

^2^ Institute of Mineral Resources Research, China Metallurgical Geology Bureau, Beijing 100025, China

^3^ Department of Geosciences and Geography, University of Helsinki, Helsinki 00014, Finland

* Email: yi.lin@pku.edu.cn

***Supplementary Tables***

| Table S1. Performance statistics based on the derived crown-internal (CI) parameters. | | | | | | | | | | | |  |
| --- | --- | --- | --- | --- | --- | --- | --- | --- | --- | --- | --- | --- |
|  |  |  |  |  |  |  |  |  |  |  |  |  |
| Sum of square between classes(B) Matrix | | | | |  |  |  |  |  |  |  |  |
| 0.0074 | 0.0082 | 0.0154 | -0.0121 | 0.0326 | 0.0168 | 0.0144 | 0.008 | -0.0072 | -0.0241 | -0.0026 | -0.0087 | -0.358 |
| 0.0082 | 0.0173 | 0.0504 | 0.0081 | 0.0118 | 0.0294 | 0.0184 | -0.0105 | 0.0007 | -0.0132 | 0.0016 | 0.0059 | -1.4802 |
| 0.0154 | 0.0504 | 0.1754 | 0.0547 | -0.005 | 0.0772 | 0.0441 | -0.0563 | 0.02 | 0.0043 | 0.0135 | 0.0447 | -4.7736 |
| -0.0121 | 0.0081 | 0.0547 | 0.0906 | -0.1592 | 0.0028 | -0.0237 | -0.0754 | 0.0366 | 0.0796 | 0.0156 | 0.0584 | -2.8894 |
| 0.0326 | 0.0118 | -0.005 | -0.1592 | 0.3427 | 0.0357 | 0.076 | 0.1261 | -0.0626 | -0.1578 | -0.0231 | -0.0929 | 3.5307 |
| 0.0168 | 0.0294 | 0.0772 | 0.0028 | 0.0357 | 0.0525 | 0.0348 | -0.0089 | -0.0047 | -0.0364 | -0.0002 | 0.001 | -2.3254 |
| 0.0144 | 0.0184 | 0.0441 | -0.0237 | 0.076 | 0.0348 | 0.0318 | 0.015 | -0.0122 | -0.0445 | -0.0035 | -0.0135 | -0.7222 |
| 0.008 | -0.0105 | -0.0563 | -0.0754 | 0.1261 | -0.0089 | 0.015 | 0.0636 | -0.03 | -0.0619 | -0.0131 | -0.0489 | 2.6748 |
| -0.0072 | 0.0007 | 0.02 | 0.0366 | -0.0626 | -0.0047 | -0.0122 | -0.03 | 0.0167 | 0.0388 | 0.0073 | 0.0257 | -0.8798 |
| -0.0241 | -0.0132 | 0.0043 | 0.0796 | -0.1578 | -0.0364 | -0.0445 | -0.0619 | 0.0388 | 0.1031 | 0.016 | 0.0557 | -0.8077 |
| -0.0026 | 0.0016 | 0.0135 | 0.0156 | -0.0231 | -0.0002 | -0.0035 | -0.0131 | 0.0073 | 0.016 | 0.0035 | 0.0117 | -0.4412 |
| -0.0087 | 0.0059 | 0.0447 | 0.0584 | -0.0929 | 0.001 | -0.0135 | -0.0489 | 0.0257 | 0.0557 | 0.0117 | 0.041 | -1.7684 |
| -0.358 | -1.4802 | -4.7736 | -2.8894 | 3.5307 | -2.3254 | -0.7222 | 2.6748 | -0.8798 | -0.8077 | -0.4412 | -1.7684 | 188.6813 |
|  |  |  |  |  |  |  |  |  |  |  |  |  |
| Sums of square within block Matrix | | | |  |  |  |  |  |  |  |  |  |
| 0.0076 | 0.0003 | 0.0082 | -0.0038 | 0.0136 | 0.0011 | 0 | 0.0014 | -0.0038 | -0.0099 | -0.0059 | -0.0202 | 0.1577 |
| 0.0003 | 0.0166 | 0.0302 | 0.0017 | 0.0112 | 0.0193 | 0.0036 | -0.0082 | 0.005 | -0.0215 | 0.0088 | 0.0305 | -0.7037 |
| 0.0082 | 0.0302 | 0.6926 | 0.0009 | 0.0121 | 0.0558 | -0.0206 | -0.2644 | 0.0361 | -0.0478 | 0.0002 | 0.0398 | -2.4819 |
| -0.0038 | 0.0017 | 0.0009 | 0.0419 | -0.0404 | 0.0018 | -0.0112 | -0.0253 | 0.016 | 0.0141 | 0.0043 | 0.05 | -0.854 |
| 0.0136 | 0.0112 | 0.0121 | -0.0404 | 0.6251 | 0.035 | 0.0415 | 0.0556 | -0.0371 | -0.0224 | 0.0103 | -0.0821 | 6.0366 |
| 0.0011 | 0.0193 | 0.0558 | 0.0018 | 0.035 | 0.0545 | 0.0026 | -0.02 | 0.0029 | -0.0308 | 0.009 | 0.0109 | -1.7129 |
| 0 | 0.0036 | -0.0206 | -0.0112 | 0.0415 | 0.0026 | 0.0208 | 0.0266 | -0.0041 | 0.0089 | 0.0051 | -0.0194 | 0.5339 |
| 0.0014 | -0.0082 | -0.2644 | -0.0253 | 0.0556 | -0.02 | 0.0266 | 0.1531 | -0.0283 | 0.0167 | -0.0005 | -0.0654 | 1.8774 |
| -0.0038 | 0.005 | 0.0361 | 0.016 | -0.0371 | 0.0029 | -0.0041 | -0.0283 | 0.0181 | 0.0305 | 0.0063 | 0.0601 | -0.7054 |
| -0.0099 | -0.0215 | -0.0478 | 0.0141 | -0.0224 | -0.0308 | 0.0089 | 0.0167 | 0.0305 | 0.3278 | 0.0026 | 0.0707 | 0.5862 |
| -0.0059 | 0.0088 | 0.0002 | 0.0043 | 0.0103 | 0.009 | 0.0051 | -0.0005 | 0.0063 | 0.0026 | 0.0147 | 0.0634 | -0.2368 |
| -0.0202 | 0.0305 | 0.0398 | 0.05 | -0.0821 | 0.0109 | -0.0194 | -0.0654 | 0.0601 | 0.0707 | 0.0634 | 0.7261 | -1.6666 |
| 0.1577 | -0.7037 | -2.4819 | -0.854 | 6.0366 | -1.7129 | 0.5339 | 1.8774 | -0.7054 | 0.5862 | -0.2368 | -1.6666 | 177.9524 |
|  |  |  |  |  |  |  |  |  |  |  |  |  |
| Total sum of square(T) Matrix | | | |  |  |  |  |  |  |  |  |  |
| 0.0149 | 0.0085 | 0.0236 | -0.0159 | 0.0462 | 0.0179 | 0.0143 | 0.0094 | -0.011 | -0.034 | -0.0085 | -0.0289 | -0.2003 |
| 0.0085 | 0.0339 | 0.0806 | 0.0098 | 0.0229 | 0.0487 | 0.022 | -0.0187 | 0.0057 | -0.0347 | 0.0103 | 0.0364 | -2.1839 |
| 0.0236 | 0.0806 | 0.868 | 0.0555 | 0.0072 | 0.133 | 0.0236 | -0.3207 | 0.0562 | -0.0435 | 0.0137 | 0.0845 | -7.2555 |
| -0.0159 | 0.0098 | 0.0555 | 0.1326 | -0.1996 | 0.0046 | -0.0348 | -0.1007 | 0.0526 | 0.0937 | 0.0199 | 0.1084 | -3.7433 |
| 0.0462 | 0.0229 | 0.0072 | -0.1996 | 0.9678 | 0.0707 | 0.1175 | 0.1817 | -0.0997 | -0.1802 | -0.0128 | -0.1751 | 9.5673 |
| 0.0179 | 0.0487 | 0.133 | 0.0046 | 0.0707 | 0.107 | 0.0374 | -0.0288 | -0.0018 | -0.0672 | 0.0088 | 0.0119 | -4.0383 |
| 0.0143 | 0.022 | 0.0236 | -0.0348 | 0.1175 | 0.0374 | 0.0526 | 0.0416 | -0.0163 | -0.0356 | 0.0016 | -0.0329 | -0.1884 |
| 0.0094 | -0.0187 | -0.3207 | -0.1007 | 0.1817 | -0.0288 | 0.0416 | 0.2168 | -0.0583 | -0.0452 | -0.0137 | -0.1144 | 4.5522 |
| -0.011 | 0.0057 | 0.0562 | 0.0526 | -0.0997 | -0.0018 | -0.0163 | -0.0583 | 0.0347 | 0.0693 | 0.0136 | 0.0857 | -1.5852 |
| -0.034 | -0.0347 | -0.0435 | 0.0937 | -0.1802 | -0.0672 | -0.0356 | -0.0452 | 0.0693 | 0.4309 | 0.0187 | 0.1264 | -0.2216 |
| -0.0085 | 0.0103 | 0.0137 | 0.0199 | -0.0128 | 0.0088 | 0.0016 | -0.0137 | 0.0136 | 0.0187 | 0.0181 | 0.0751 | -0.6781 |
| -0.0289 | 0.0364 | 0.0845 | 0.1084 | -0.1751 | 0.0119 | -0.0329 | -0.1144 | 0.0857 | 0.1264 | 0.0751 | 0.767 | -3.4351 |
| -0.2003 | -2.1839 | -7.2555 | -3.7433 | 9.5673 | -4.0383 | -0.1884 | 4.5522 | -1.5852 | -0.2216 | -0.6781 | -3.4351 | 366.6336 |
| Wilks Statistics=0.028719 | | |  |  |  |  |  |  |  |  |  |  |
| Degree of freeness=(13,36,3) | | | |  |  |  |  |  |  |  |  |  |
| Chi-Square value=108.281 | | | p=0.00010 | |  |  |  |  |  |  |  |  |

Table S2. Tree species classification based on the CI structure parameters and the SVM-LOOCV classification algorithm (κ=0.7643).

| Case | Species | Precision (%) | Recall (%) | Accuracy (%) |
| --- | --- | --- | --- | --- |
| Optimal | PA | 100.00 | 100.00 | 82.50 |
|  | PS | 100.00 | 85.71 |  |
|  | PT | 55.56 | 71.43 |  |
|  | QR | 70.00 | 70.00 |  |

| Table S3. Performance statistics based on the derived tree-external (TE) parameters. | | | | | | | | | | |
| --- | --- | --- | --- | --- | --- | --- | --- | --- | --- | --- |
|  |  |  |  |  |  |  |  |  |  |  |
| Sum of square between classes(B) Matrix | | | | |  |  |  |  |  |  |
| 0.9242 | 0.014 | 3.9103 | -0.7052 | 0.0012 | 0.3049 | -0.5651 | 6.719 | 3.8438 | 0.5374 | 0.271 |
| 0.014 | 0.2183 | -1.6777 | -0.0886 | 0.052 | 0.0353 | -0.0172 | -0.2281 | -3.1575 | 3.5666 | 10.2907 |
| 3.9103 | -1.6777 | 30.3845 | -2.3775 | -0.4271 | 1.0478 | -2.3221 | 31.094 | 42.4272 | -25.843 | -81.3689 |
| -0.7052 | -0.0886 | -2.3775 | 0.6004 | 0.0259 | -0.2505 | 0.4354 | -5.1077 | -3.1493 | -2.242 | -2.4421 |
| 0.0012 | 0.052 | -0.4271 | 0.0259 | 0.0723 | -0.0013 | -0.0015 | -0.199 | -2.5623 | 0.109 | 4.3516 |
| 0.3049 | 0.0353 | 1.0478 | -0.2505 | -0.0013 | 0.1063 | -0.1878 | 2.1895 | 1.0858 | 0.7903 | 1.2536 |
| -0.5651 | -0.0172 | -2.3221 | 0.4354 | -0.0015 | -0.1878 | 0.3459 | -4.0982 | -2.262 | -0.4871 | -0.5341 |
| 6.719 | -0.2281 | 31.094 | -5.1077 | -0.199 | 2.1895 | -4.0982 | 49.6273 | 36.6921 | 0.1256 | -17.6756 |
| 3.8438 | -3.1575 | 42.4272 | -3.1493 | -2.5623 | 1.0858 | -2.262 | 36.6921 | 117.5541 | -28.0024 | -207.691 |
| 0.5374 | 3.5666 | -25.843 | -2.242 | 0.109 | 0.7903 | -0.4871 | 0.1256 | -28.0024 | 67.5183 | 144.5716 |
| 0.271 | 10.2907 | -81.3689 | -2.4421 | 4.3516 | 1.2536 | -0.5341 | -17.6756 | -207.691 | 144.5716 | 545.6245 |
|  |  |  |  |  |  |  |  |  |  |  |
| Sums of square within block Matrix | | | |  |  |  |  |  |  |  |
| 0.6753 | 0.0962 | 1.6399 | -0.0573 | -0.0373 | 0.0508 | -0.271 | 1.8132 | -1.8784 | 1.1634 | -5.6569 |
| 0.0962 | 0.1873 | -0.8264 | -0.0038 | -0.0341 | -0.0003 | -0.0337 | 0.095 | -1.3478 | 3.2004 | -3.2905 |
| 1.6399 | -0.8264 | 13.0476 | -0.0528 | 0.1836 | 0.1533 | -0.6709 | 5.6378 | 6.8241 | -16.3228 | -2.5235 |
| -0.0573 | -0.0038 | -0.0528 | 0.6096 | -0.1151 | -0.0735 | 0.0689 | -0.7083 | -3.0822 | -1.1431 | -0.4862 |
| -0.0373 | -0.0341 | 0.1836 | -0.1151 | 0.668 | -0.0449 | 0.0511 | -0.7309 | -2.411 | -1.5505 | -2.4787 |
| 0.0508 | -0.0003 | 0.1533 | -0.0735 | -0.0449 | 0.1352 | -0.1232 | 0.7849 | 1.1845 | 0.3361 | -3.8333 |
| -0.271 | -0.0337 | -0.6709 | 0.0689 | 0.0511 | -0.1232 | 0.1969 | -1.231 | -0.0323 | -0.5822 | 4.8808 |
| 1.8132 | 0.095 | 5.6378 | -0.7083 | -0.7309 | 0.7849 | -1.231 | 19.9648 | 27.1725 | 12.4438 | -18.8366 |
| -1.8784 | -1.3478 | 6.8241 | -3.0822 | -2.411 | 1.1845 | -0.0323 | 27.1725 | 260.8793 | 61.9026 | 27.534 |
| 1.1634 | 3.2004 | -16.3228 | -1.1431 | -1.5505 | 0.3361 | -0.5822 | 12.4438 | 61.9026 | 88.4855 | -37.287 |
| -5.6569 | -3.2905 | -2.5235 | -0.4862 | -2.4787 | -3.8333 | 4.8808 | -18.8366 | 27.534 | -37.287 | 881.4602 |
|  |  |  |  |  |  |  |  |  |  |  |
| Total sum of square(T) Matrix | | | |  |  |  |  |  |  |  |
| 1.5995 | 0.1101 | 5.5502 | -0.7625 | -0.0361 | 0.3556 | -0.836 | 8.5322 | 1.9654 | 1.7009 | -5.3859 |
| 0.1101 | 0.4055 | -2.5041 | -0.0924 | 0.0178 | 0.0351 | -0.0509 | -0.133 | -4.5053 | 6.767 | 7.0003 |
| 5.5502 | -2.5041 | 43.4321 | -2.4303 | -0.2435 | 1.2012 | -2.993 | 36.7319 | 49.2512 | -42.1658 | -83.8925 |
| -0.7625 | -0.0924 | -2.4303 | 1.21 | -0.0892 | -0.324 | 0.5043 | -5.816 | -6.2315 | -3.3851 | -2.9283 |
| -0.0361 | 0.0178 | -0.2435 | -0.0892 | 0.7402 | -0.0462 | 0.0496 | -0.9299 | -4.9733 | -1.4416 | 1.8729 |
| 0.3556 | 0.0351 | 1.2012 | -0.324 | -0.0462 | 0.2414 | -0.311 | 2.9743 | 2.2702 | 1.1264 | -2.5798 |
| -0.836 | -0.0509 | -2.993 | 0.5043 | 0.0496 | -0.311 | 0.5428 | -5.3292 | -2.2943 | -1.0693 | 4.3467 |
| 8.5322 | -0.133 | 36.7319 | -5.816 | -0.9299 | 2.9743 | -5.3292 | 69.5921 | 63.8646 | 12.5694 | -36.5122 |
| 1.9654 | -4.5053 | 49.2512 | -6.2315 | -4.9733 | 2.2702 | -2.2943 | 63.8646 | 378.4334 | 33.9002 | -180.157 |
| 1.7009 | 6.767 | -42.1658 | -3.3851 | -1.4416 | 1.1264 | -1.0693 | 12.5694 | 33.9002 | 156.0038 | 107.2846 |
| -5.3859 | 7.0003 | -83.8925 | -2.9283 | 1.8729 | -2.5798 | 4.3467 | -36.5122 | -180.157 | 107.2846 | 1427.085 |
| Wilks Statistics=0.027113 | | |  |  |  |  |  |  |  |  |
| Degree of freeness=(11,36,3) | | | |  |  |  |  |  |  |  |
| Chi-Square value=113.644 | | | p=0.00010 | |  |  |  |  |  |  |

Table S4. Tree species classification based on the TE feature parameters and the SVM-LOOCV classification algorithm (κ=0.7936).

| Case | Species | Precision (%) | Recall (%) | Accuracy (%) |
| --- | --- | --- | --- | --- |
| Optimal | PA | 88.89 | 88.89 | 85.00 |
|  | PS | 81.25 | 92.86 |  |
|  | PT | 66.67 | 57.14 |  |
|  | QR | 100.00 | 90.00 |  |

| Table S5. Performance statistics based on the derived optimal CI and TE parameters. | | | | | | | | | |
| --- | --- | --- | --- | --- | --- | --- | --- | --- | --- |
|  |  |  |  |  |  |  |  |  |  |
| Sum of square between classes(B) Matrix | | | | |  |  |  |  |  |
| 0.9242 | 0.014 | 3.9103 | -0.7052 | 0.0012 | 0.3049 | -0.5651 | 6.719 | 3.8438 | 0.5374 |
| 0.014 | 0.2183 | -1.6777 | -0.0886 | 0.052 | 0.0353 | -0.0172 | -0.2281 | -3.1575 | 3.5666 |
| 3.9103 | -1.6777 | 30.3845 | -2.3775 | -0.4271 | 1.0478 | -2.3221 | 31.094 | 42.4272 | -25.843 |
| -0.7052 | -0.0886 | -2.3775 | 0.6004 | 0.0259 | -0.2505 | 0.4354 | -5.1077 | -3.1493 | -2.242 |
| 0.0012 | 0.052 | -0.4271 | 0.0259 | 0.0723 | -0.0013 | -0.0015 | -0.199 | -2.5623 | 0.109 |
| 0.3049 | 0.0353 | 1.0478 | -0.2505 | -0.0013 | 0.1063 | -0.1878 | 2.1895 | 1.0858 | 0.7903 |
| -0.5651 | -0.0172 | -2.3221 | 0.4354 | -0.0015 | -0.1878 | 0.3459 | -4.0982 | -2.262 | -0.4871 |
| 6.719 | -0.2281 | 31.094 | -5.1077 | -0.199 | 2.1895 | -4.0982 | 49.6273 | 36.6921 | 0.1256 |
| 3.8438 | -3.1575 | 42.4272 | -3.1493 | -2.5623 | 1.0858 | -2.262 | 36.6921 | 117.5541 | -28.0024 |
| 0.5374 | 3.5666 | -25.843 | -2.242 | 0.109 | 0.7903 | -0.4871 | 0.1256 | -28.0024 | 67.5183 |
|  |  |  |  |  |  |  |  |  |  |
| Sums of square within block Matrix | | | |  |  |  |  |  |  |
| 0.6753 | 0.0962 | 1.6399 | -0.0573 | -0.0373 | 0.0508 | -0.271 | 1.8132 | -1.8784 | 1.1634 |
| 0.0962 | 0.1873 | -0.8264 | -0.0038 | -0.0341 | -0.0003 | -0.0337 | 0.095 | -1.3478 | 3.2004 |
| 1.6399 | -0.8264 | 13.0476 | -0.0528 | 0.1836 | 0.1533 | -0.6709 | 5.6378 | 6.8241 | -16.3228 |
| -0.0573 | -0.0038 | -0.0528 | 0.6096 | -0.1151 | -0.0735 | 0.0689 | -0.7083 | -3.0822 | -1.1431 |
| -0.0373 | -0.0341 | 0.1836 | -0.1151 | 0.668 | -0.0449 | 0.0511 | -0.7309 | -2.411 | -1.5505 |
| 0.0508 | -0.0003 | 0.1533 | -0.0735 | -0.0449 | 0.1352 | -0.1232 | 0.7849 | 1.1845 | 0.3361 |
| -0.271 | -0.0337 | -0.6709 | 0.0689 | 0.0511 | -0.1232 | 0.1969 | -1.231 | -0.0323 | -0.5822 |
| 1.8132 | 0.095 | 5.6378 | -0.7083 | -0.7309 | 0.7849 | -1.231 | 19.9648 | 27.1725 | 12.4438 |
| -1.8784 | -1.3478 | 6.8241 | -3.0822 | -2.411 | 1.1845 | -0.0323 | 27.1725 | 260.8793 | 61.9026 |
| 1.1634 | 3.2004 | -16.3228 | -1.1431 | -1.5505 | 0.3361 | -0.5822 | 12.4438 | 61.9026 | 88.4855 |
|  |  |  |  |  |  |  |  |  |  |
| Total sum of square(T) Matrix | | | |  |  |  |  |  |  |
| 1.5995 | 0.1101 | 5.5502 | -0.7625 | -0.0361 | 0.3556 | -0.836 | 8.5322 | 1.9654 | 1.7009 |
| 0.1101 | 0.4055 | -2.5041 | -0.0924 | 0.0178 | 0.0351 | -0.0509 | -0.133 | -4.5053 | 6.767 |
| 5.5502 | -2.5041 | 43.4321 | -2.4303 | -0.2435 | 1.2012 | -2.993 | 36.7319 | 49.2512 | -42.1658 |
| -0.7625 | -0.0924 | -2.4303 | 1.21 | -0.0892 | -0.324 | 0.5043 | -5.816 | -6.2315 | -3.3851 |
| -0.0361 | 0.0178 | -0.2435 | -0.0892 | 0.7402 | -0.0462 | 0.0496 | -0.9299 | -4.9733 | -1.4416 |
| 0.3556 | 0.0351 | 1.2012 | -0.324 | -0.0462 | 0.2414 | -0.311 | 2.9743 | 2.2702 | 1.1264 |
| -0.836 | -0.0509 | -2.993 | 0.5043 | 0.0496 | -0.311 | 0.5428 | -5.3292 | -2.2943 | -1.0693 |
| 8.5322 | -0.133 | 36.7319 | -5.816 | -0.9299 | 2.9743 | -5.3292 | 69.5921 | 63.8646 | 12.5694 |
| 1.9654 | -4.5053 | 49.2512 | -6.2315 | -4.9733 | 2.2702 | -2.2943 | 63.8646 | 378.4334 | 33.9002 |
| 1.7009 | 6.767 | -42.1658 | -3.3851 | -1.4416 | 1.1264 | -1.0693 | 12.5694 | 33.9002 | 156.0038 |
| Wilks Statistic=0.048847 | | |  |  |  |  |  |  |  |
| Degree of freeness=(10,36,3) | | | |  |  |  |  |  |  |
| Chi-Square value=96.610 | | | p=0.00010 | |  |  |  |  |  |

Table S6. Tree species classification based on the CI and TE feature parameters and the SVM-LOOCV classification algorithm (κ=0.7936).

| Case | Species | Precision (%) | Recall (%) | Accuracy (%) |
| --- | --- | --- | --- | --- |
| Optimal | PA | 88.89 | 88.89 | 85.00 |
|  | PS | 81.25 | 92.86 |  |
|  | PT | 66.67 | 57.14 |  |
|  | QR | 100.00 | 90.00 |  |

***Supplementary Figures***


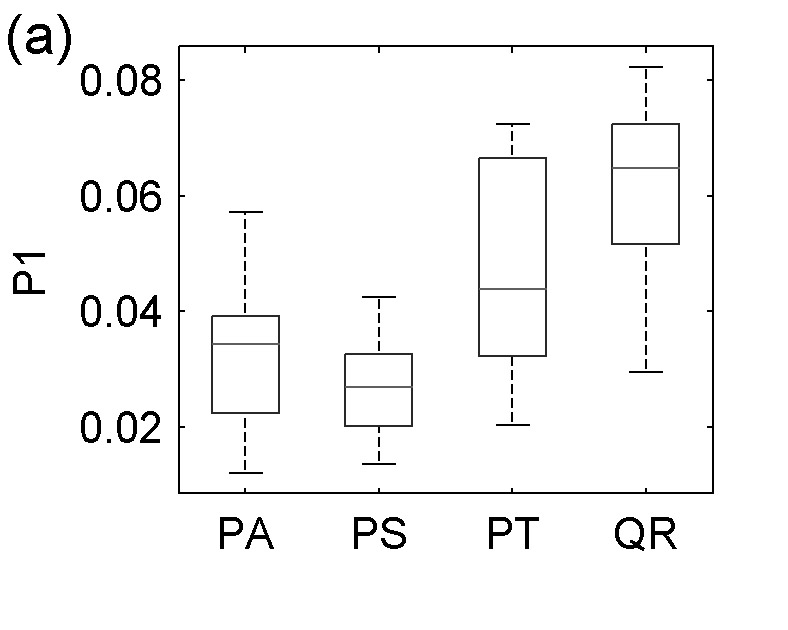

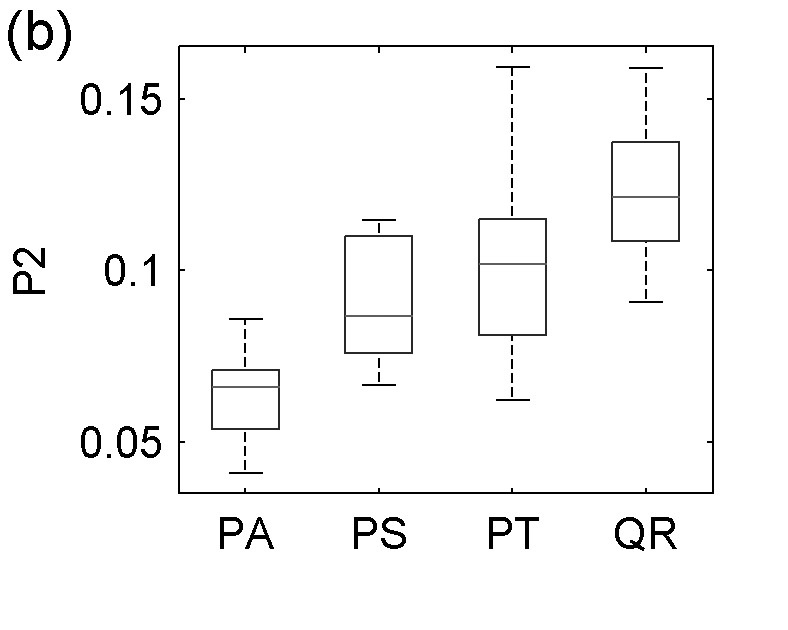

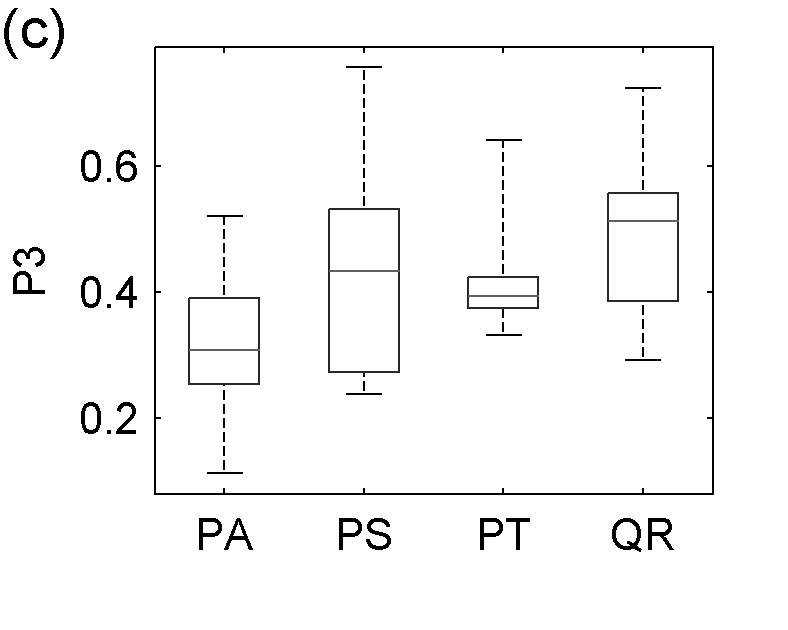

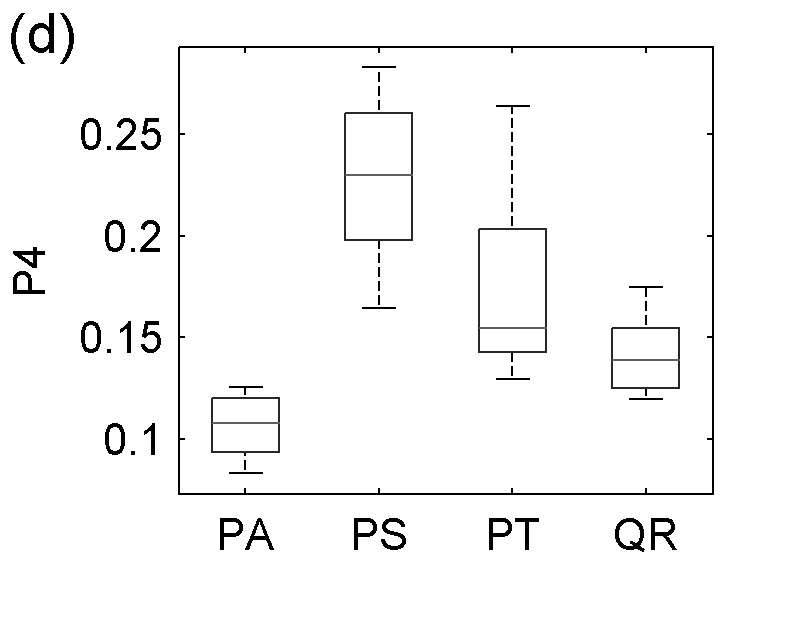

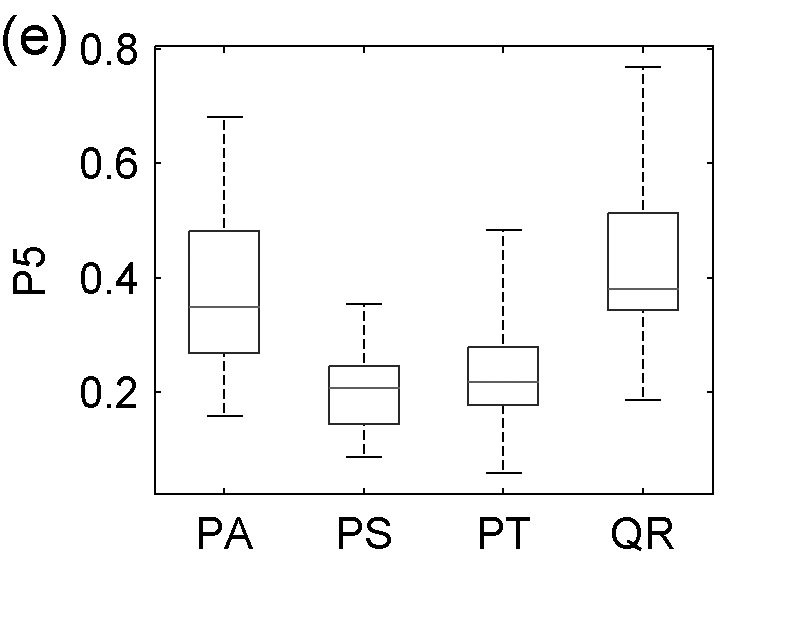

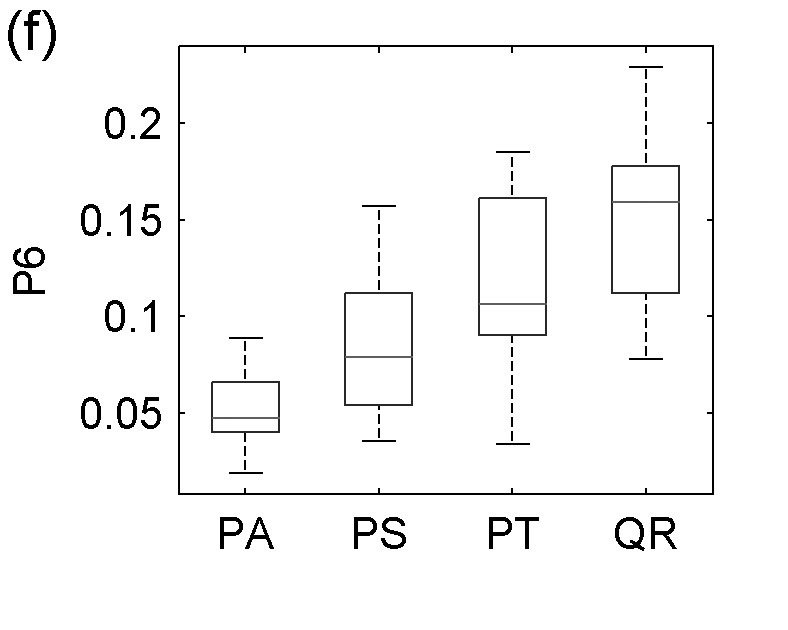

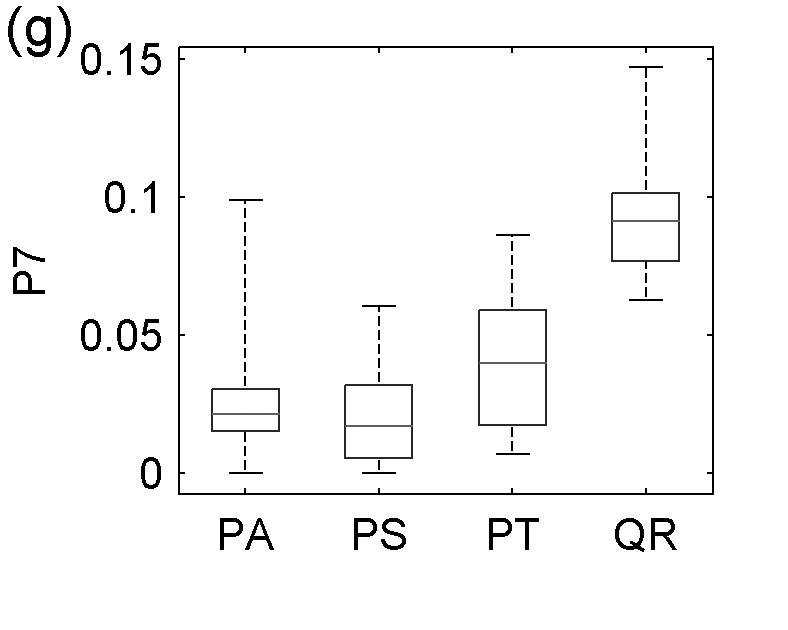

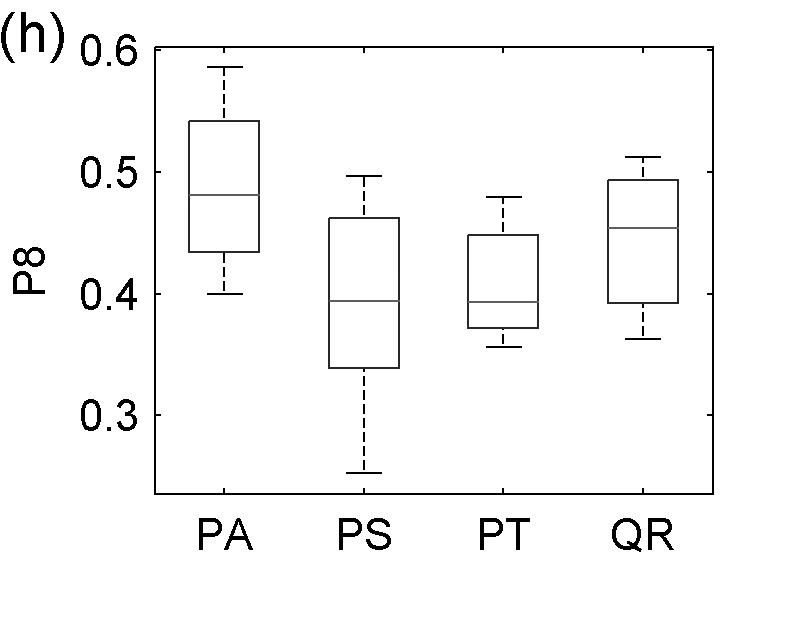

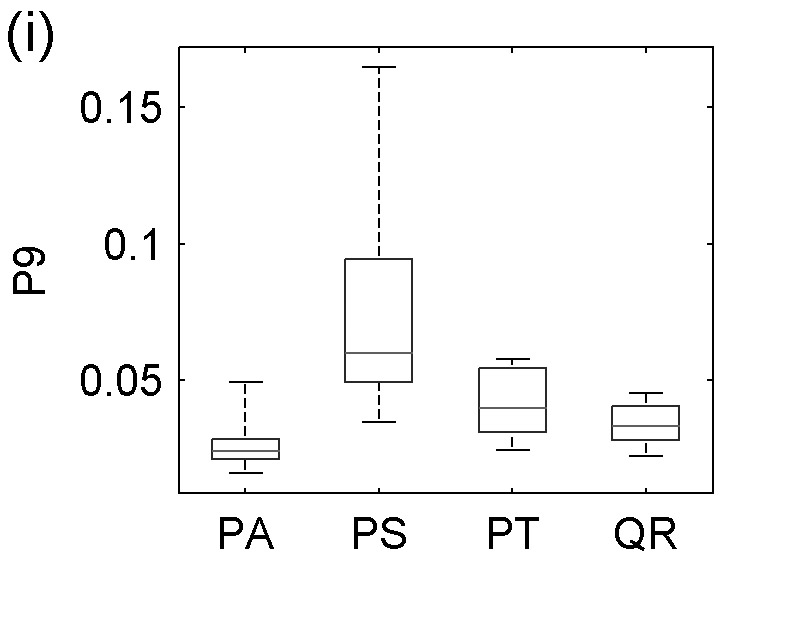

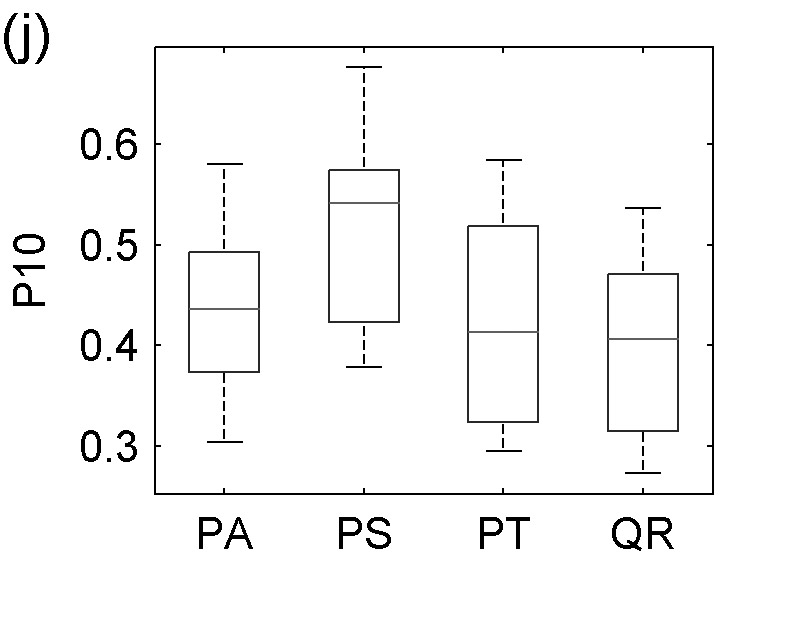

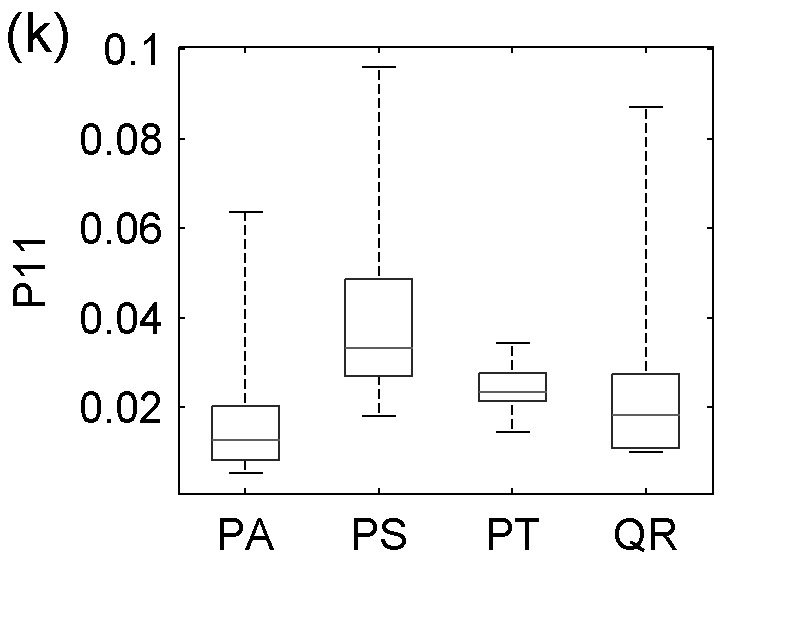

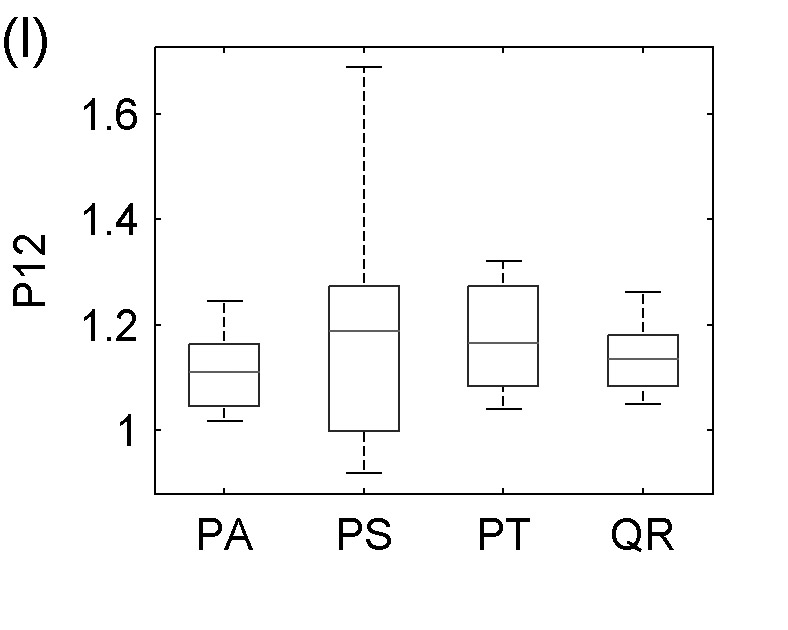

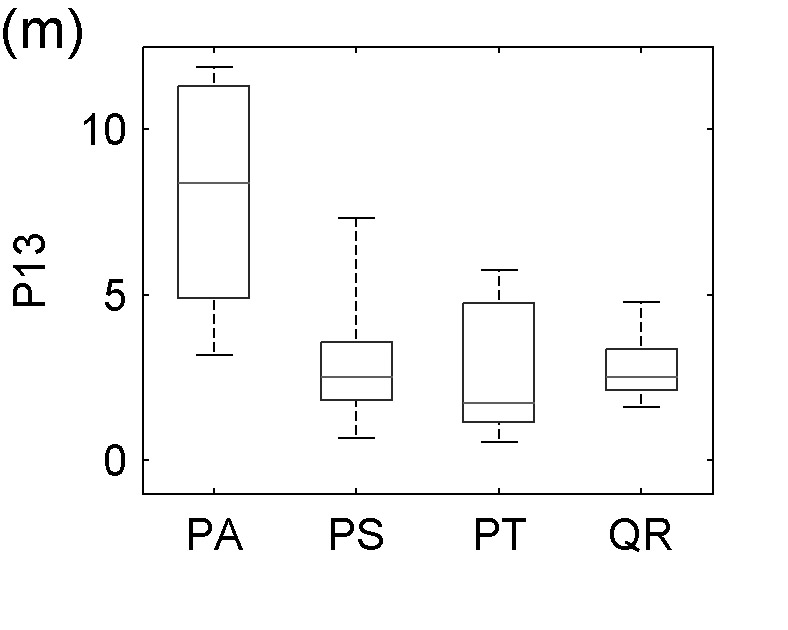


Figure S1. Boxplots of the derived values of the CI structure parameters: (a) P1, (b) P2, (c) P3, (d) P4, (e) P5, (f) P6, (g) P7, (h) P8, (i) P9, (j) P10, (k) P11, (l) P12 and (m) P13, as defined in Table 2.


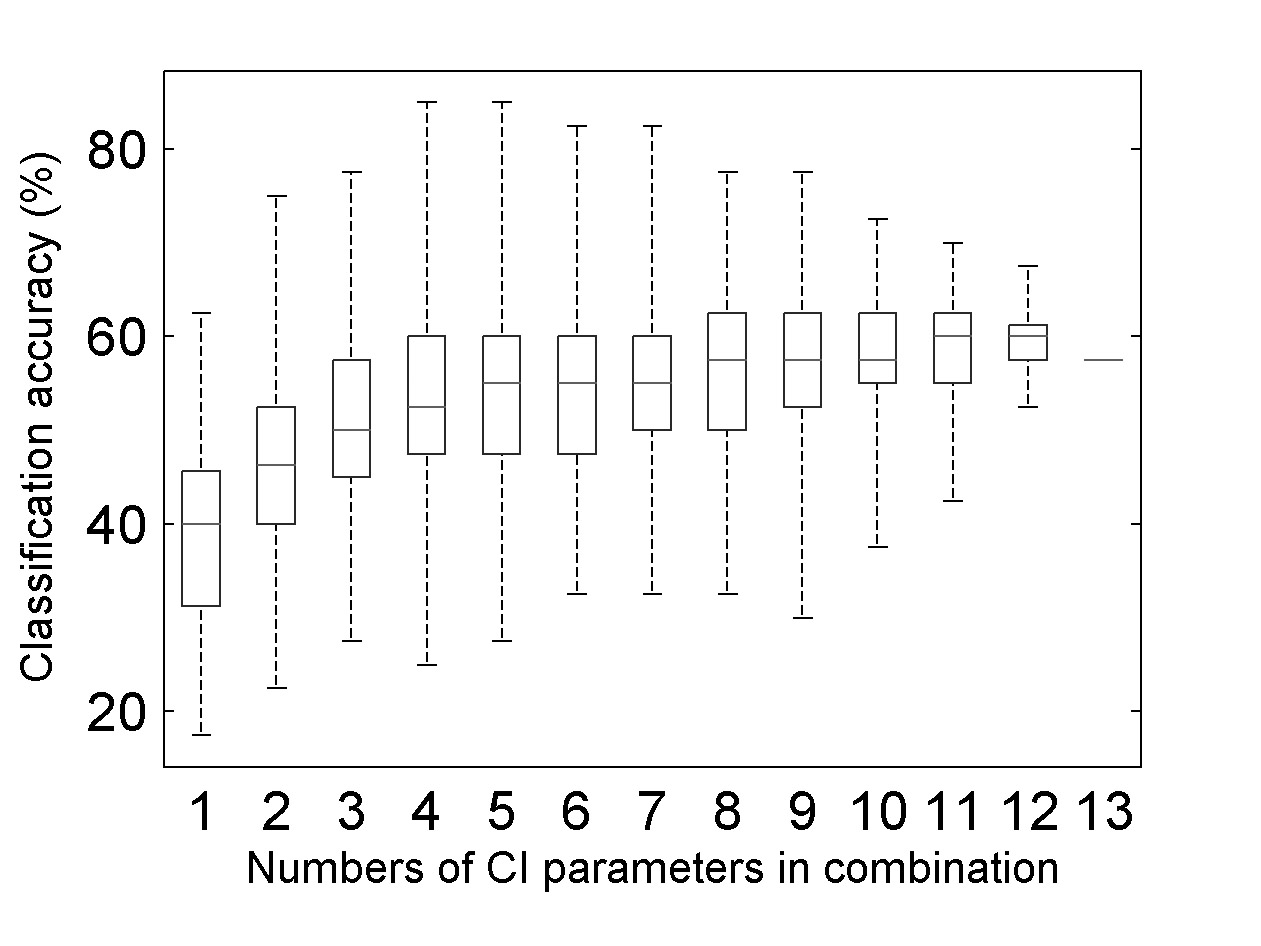


Figure S2. Boxplots of the classification accuracies for combinations of the extracted CI structure parameters with their increasing numbers.


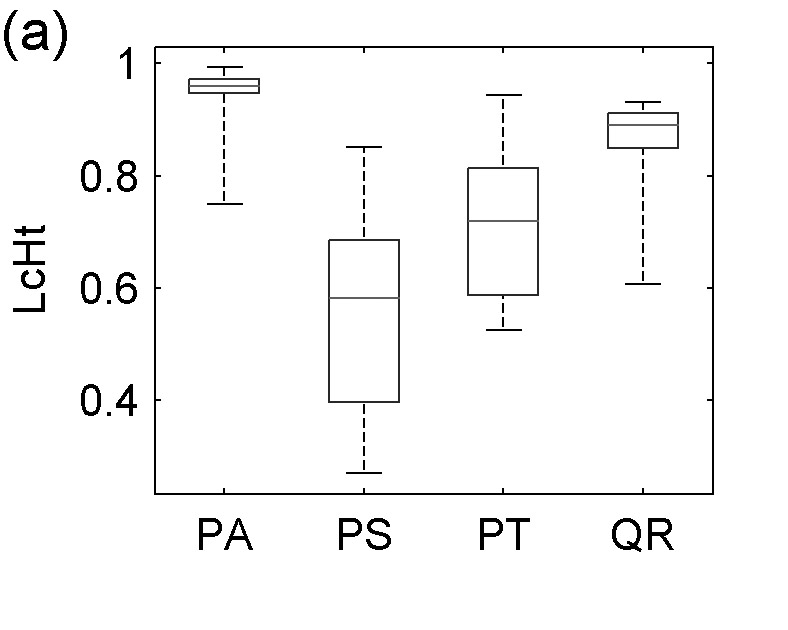

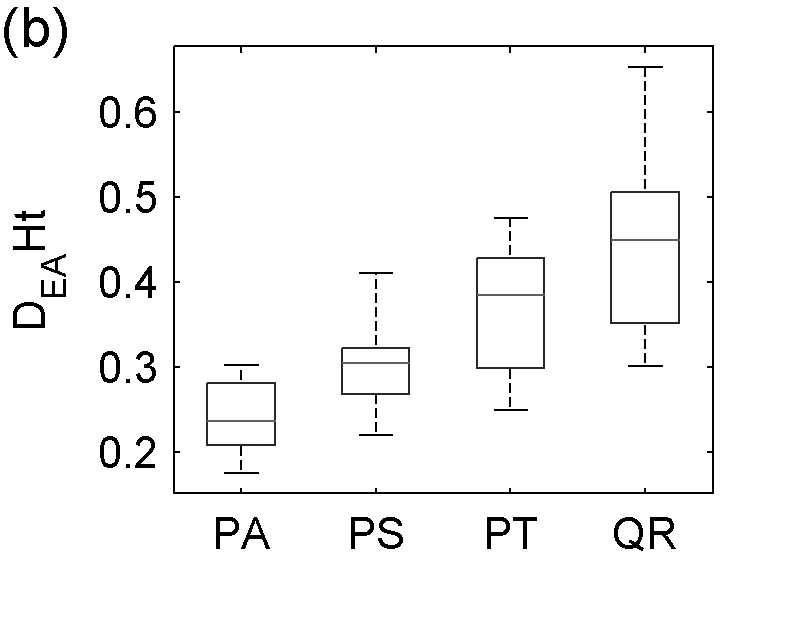

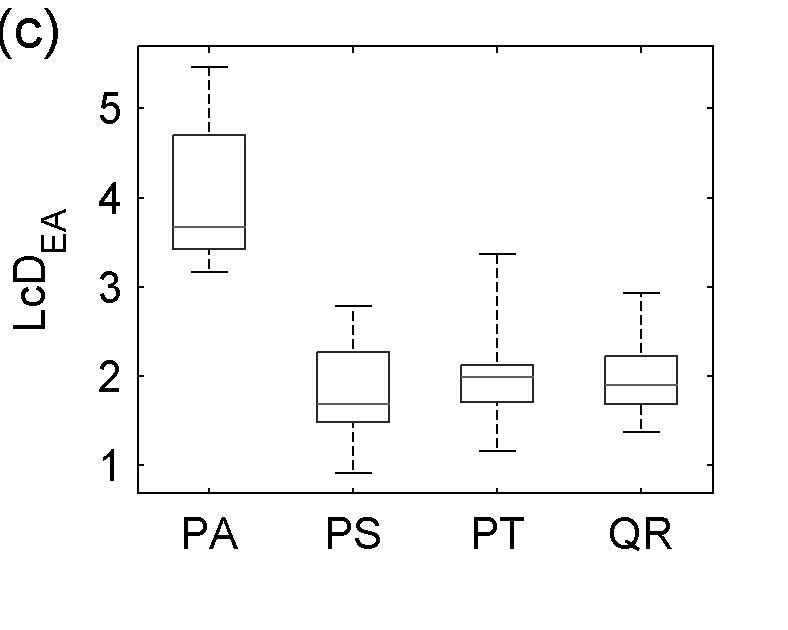

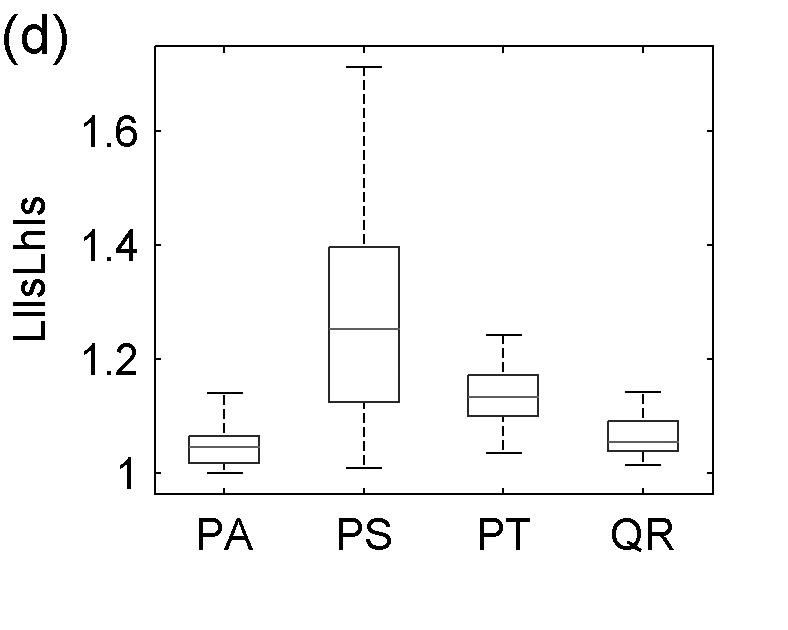

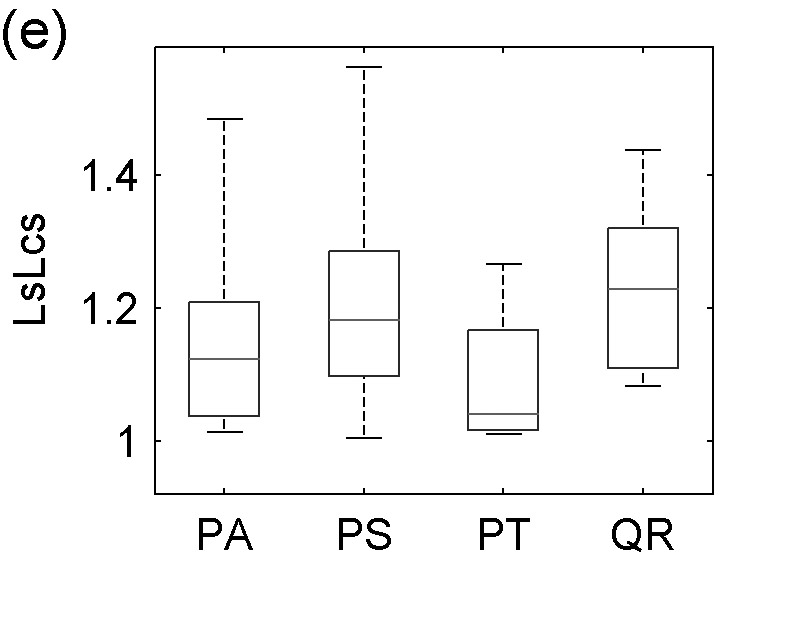

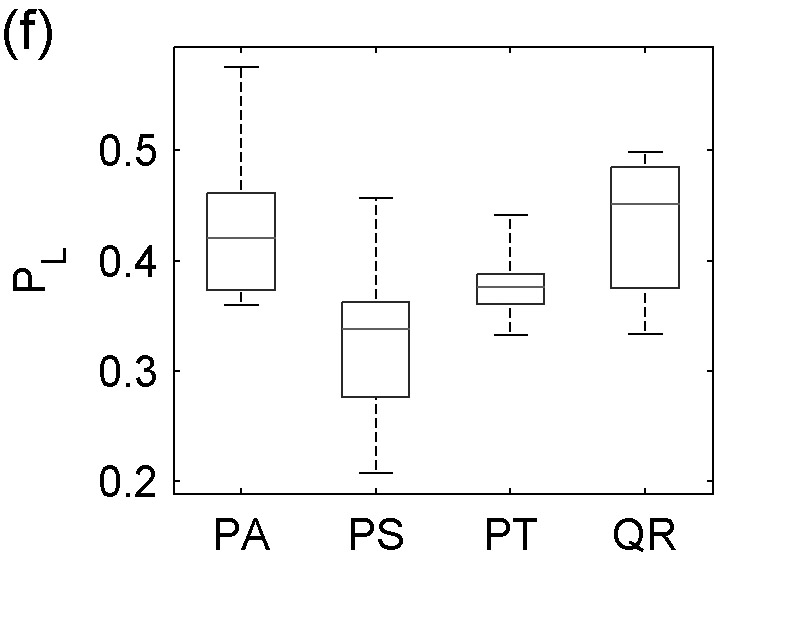

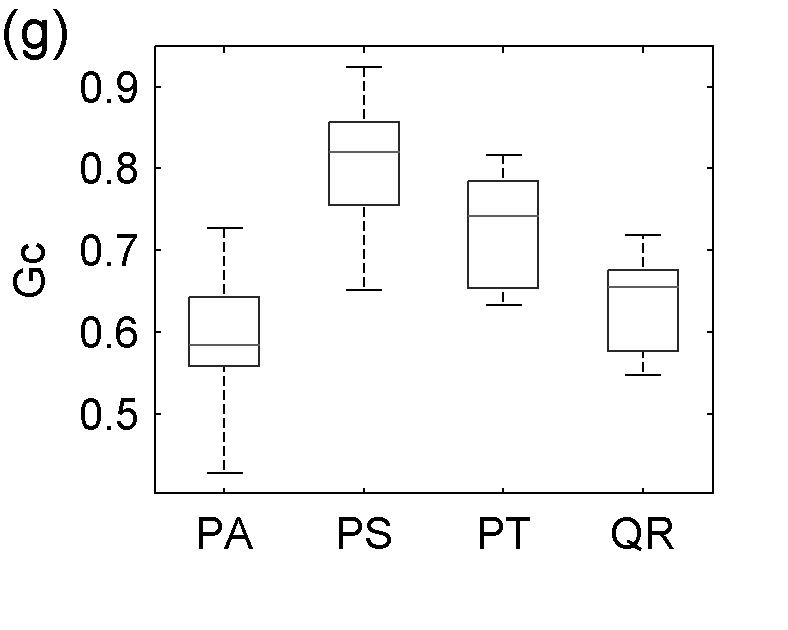

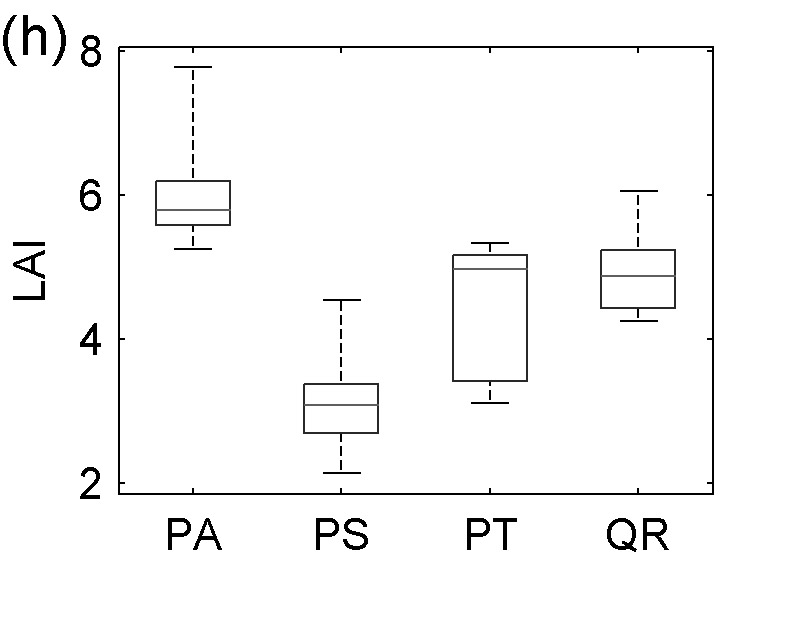

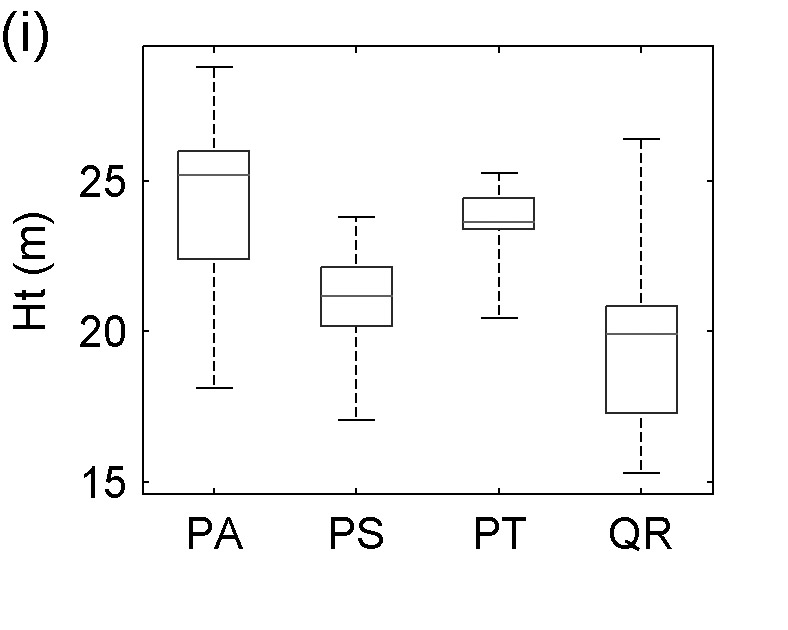

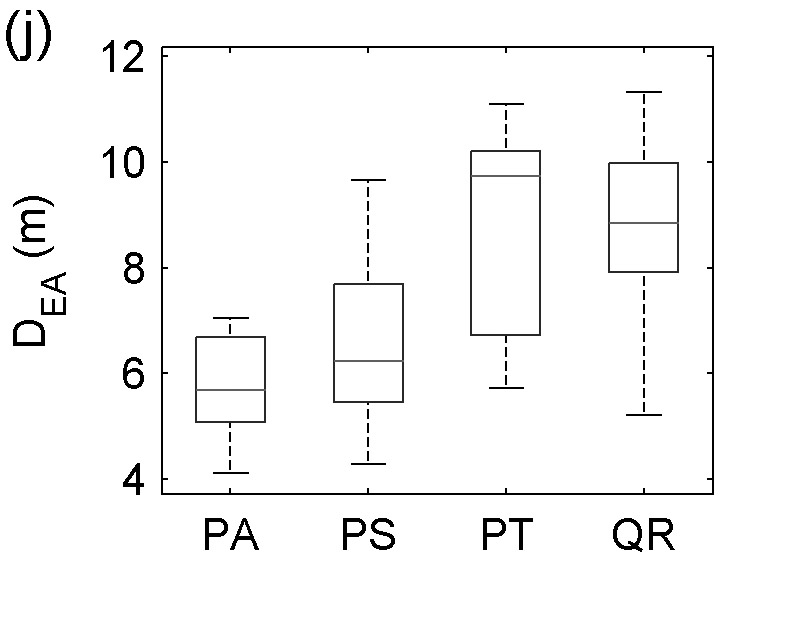

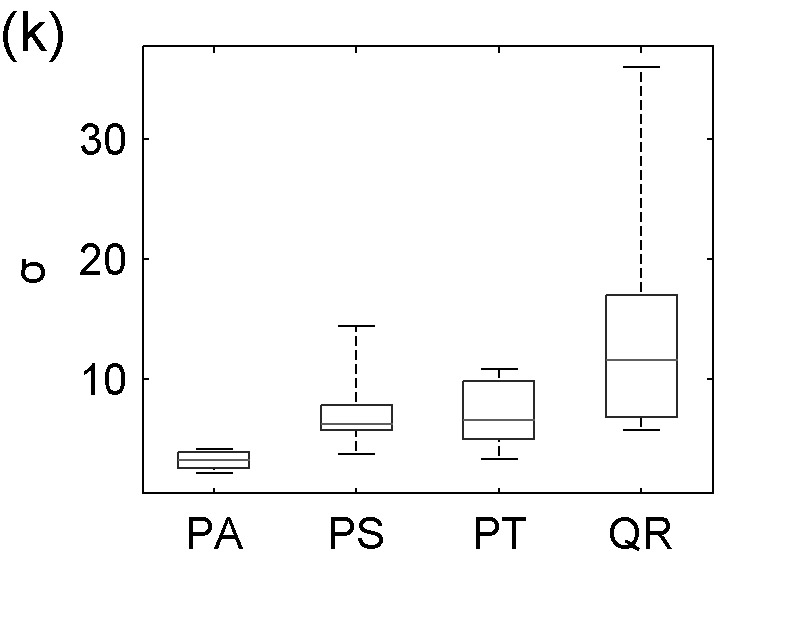


Figure S3. Boxplots of the derived values of the TE structure parameters: (a) LcHt, (b) D_EA_Ht, (c) LcD_EA_, (d) LllsLhls, (e) LsLcs, (f) P_L_, (g) Gc, (h) LAI, (i) Ht, (j) D_EA_, and (k) σ, as defined in Table 3.


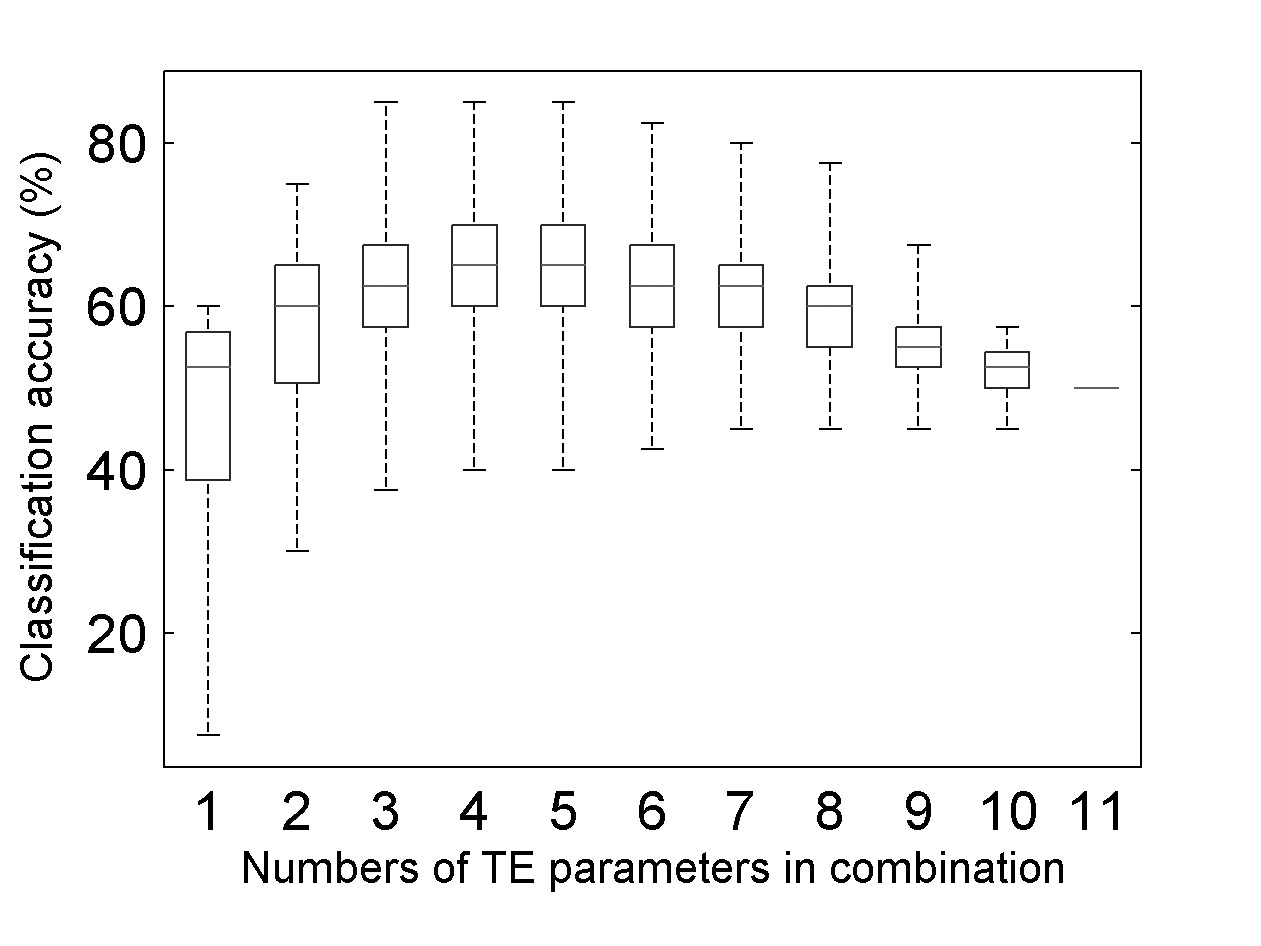


Figure S4. Boxplots of the classification accuracies for combinations of the extracted TE feature parameters with their increasing numbers.
